# Tyrosine kinase inhibitors and survival in advanced or metastatic gastric cancer: a systematic review and meta-analysis

**DOI:** 10.3389/fonc.2026.1771234

**Published:** 2026-03-24

**Authors:** Yongshan Li, Yuhui Xue, Yu Tang, Peng Zhou, Zhihui Yang, Shantong Liu, Jiazeng Xia

**Affiliations:** 1 Department of General Surgery, Institute of General Surgical Research, Jiangnan University Medical Center, School of Medicine, Jiangnan University, Wuxi, China; 2 Institute of Urology, Wuxi School of Medicine, Jiangnan University, Wuxi, China; 3 Xiangya Hospital, Central South University, Changsha, China

**Keywords:** advanced gastric cancer, meta-analysis, safety, survival, tyrosine kinase inhibitors

## Abstract

**Background:**

Small-molecule tyrosine kinase inhibitors (TKIs) have been extensively investigated in the management of advanced or metastatic gastric cancer (GC); however, their specific efficacy and optimal therapeutic role remain subjects of ongoing debate. Given the accumulation of new trial evidence and persistent uncertainty regarding patient subgroups most likely to benefit, we conducted an updated systematic review and meta-analysis to comprehensively evaluate the impact of TKIs on clinical outcomes in advanced GC.

**Methods:**

We systematically searched PubMed, Web of Science, and Embase databases for articles published up to March 1, 2026, following PRISMA 2020 guidelines. Randomized controlled trials (RCTs) comparing TKI-containing regimens versus non-TKI regimens in patients with cytologically or pathologically confirmed advanced GC were eligible. Hazard ratios (HRs) with 95% confidence intervals (CIs) were pooled for progression-free survival (PFS) and overall survival (OS) using random-effects models. Risk ratios (RRs) with 95% CIs were calculated for objective response rate (ORR) and disease control rate (DCR). Study quality was assessed using the Cochrane Risk of Bias 2 (RoB 2) tool, and evidence certainty was evaluated using the GRADE framework.

**Results:**

Ten RCTs comprising 1810 patients (1169 experimental, 641 control) met inclusion criteria. TKI-containing regimens significantly improved OS (HR 0.76, 95% CI 0.63-0.92, P = 0.005; moderate certainty) and prolonged PFS (HR 0.51, 95% CI 0.35-0.73, P = 0.0003; low certainty) compared to non-TKI regimens. DCR was markedly improved (RR 3.98, 95% CI 2.08-7.58, P < 0.0001; moderate certainty), whereas ORR did not reach statistical significance (RR 2.03, 95% CI 0.83-5.01, P = 0.12; low certainty). Subgroup analyses revealed that TKI monotherapy significantly improved both OS and PFS, whereas combination with chemotherapy did not demonstrate additive survival benefits. Grade ≥3 adverse events, including hypertension (RR 31.5, 95% CI 11.71-84.73) and hand-foot syndrome (RR 55.0, 95% CI 7.65-395.32), were more frequent with TKIs but were largely predictable and clinically manageable.

**Conclusions:**

This updated meta-analysis provides moderate-certainty evidence that TKI-containing regimens confer significant survival benefits in advanced GC, with a manageable safety profile. The differential efficacy observed between monotherapy and combination regimens underscores the need for biomarker-driven patient selection and optimization of treatment sequencing. These findings refine the current evidence base and support the continued role of TKIs in treatment algorithms for advanced GC, while highlighting critical knowledge gaps requiring further investigation.

**Registration:**

https://www.crd.york.ac.uk/prospero/display_record.php?ID=CRD42024544568, identifier CRD42024544568.

## 1 Introduction

Gastric cancer (GC) remains a significant global health burden, identified as the fifth most prevalent malignant tumor and the fourth leading cause of cancer-related mortality worldwide (1, 2). The incidence of GC exhibits marked geographical variability, with the highest rates observed in Eastern Asia (Japan and Korea) and Eastern Europe, whereas lower incidence is reported in Northern Europe, North America, and Africa (2). Despite a notable global reduction in incidence over recent decades, the majority of GC cases are diagnosed at an advanced stage, resulting in 5-year overall survival (OS) rates below 30% across all stages. Systemic chemotherapy currently constitutes the primary treatment for metastatic GC (mGC), with patients receiving conventional chemotherapy demonstrating a median overall survival (mOS) of approximately 12 months (3).

Since their emergence in the early 2000s, tyrosine kinase inhibitors (TKIs) have emerged as potent pathway-directed anticancer agents, exhibiting remarkable efficacy in various malignancies. Notably, anti-angiogenic agents that inhibit the vascular endothelial growth factor (VEGF) pathway have shown considerable promise in enhancing antitumor efficacy. Numerous clinical trials have highlighted the potential benefits of angiogenesis inhibitors for patients with GC (4–9). In a pivotal Phase III clinical study involving 273 patients receiving second-line or subsequent treatments (9), the anti-angiogenic TKI apatinib demonstrated significant improvements in median progression-free survival (mPFS) (2.6 months vs 1.8 months, P < 0.001) and disease control rate (42.05% vs 8.79%, P < 0.001) compared to placebo. Consequently, apatinib has gained regulatory approval for advanced gastric or gastro-esophageal junction (GEJ) adenocarcinoma as third-line or subsequent therapy. Similarly, regorafenib—a multi-target kinase inhibitor targeting VEGFR1/2/3 and other signaling pathways—has exhibited potent inhibition of tumor growth and angiogenesis in both preclinical and clinical studies (10), while sunitinib, an oral multi-target TKI with anti-VEGFR activity, exerts therapeutic effects through angiogenesis blockade (11).

Despite these advances, the overall effectiveness and optimal positioning of TKIs in advanced GC remain incompletely defined. Previous systematic reviews and meta-analyses have yielded inconclusive results, limited by smaller sample sizes, heterogeneous inclusion criteria, and absence of recently completed trials (12–18). Furthermore, critical questions persist regarding whether TKIs should be administered as monotherapy or in combination with chemotherapy, which patient subgroups derive greatest benefit, and how the toxicity profile compares across different treatment strategies. These knowledge gaps have important implications for clinical decision-making and future trial design.

To address these uncertainties, we conducted an updated systematic review and meta-analysis with the following objectives: (1) to provide a comprehensive, contemporary synthesis of efficacy outcomes (OS, PFS, ORR, DCR) associated with TKI-containing regimens in advanced GC; (2) to evaluate whether treatment effects differ by administration strategy (monotherapy versus combination with chemotherapy) and trial phase; (3) to systematically assess the safety profile of TKIs, including grade ≥3 adverse events; and (4) to critically appraise the quality and certainty of available evidence using standardized methodologies (Cochrane RoB 2, GRADE). This meta-analysis extends prior work by incorporating the most recent evidence through March 2026, performing rigorous subgroup analyses to explore clinical heterogeneity, and providing a structured evaluation of evidence certainty to guide clinical interpretation.

## 2 Materials and methods

### 2.1 Search strategy

We conducted a systematic review and meta-analysis to assess the efficacy and safety of TKIs in patients with advanced GC, according to PRISMA (Preferred Reporting Items for Systematic Reviews and Meta-Analysis) recommendations (PROSPERO CRD42024544568). To identify relevant articles, we comprehensively searched PubMed, EMBASE, and Web of Science databases up until October 20, 2024. After eliminating duplicate records, we screened titles and abstracts followed by a thorough evaluation of full texts. Any discrepancies regarding inclusion criteria were resolved through consensus discussion. The retrieval strategy (using PubMed as an example) is presented in **Table 1**.

**Table 1:** Literature search strategy on PubMed.

Search number	Search terms
#5	#3 AND #4
#4	Search: (((((((((Advanced Stomach Neoplasms) OR (Advanced Stomach Neoplasm)) OR (Advanced Gastric Neoplasms)) OR (Advanced Gastric Neoplasm)) OR (Advanced Cancer of Stomach)) OR (Advanced Stomach Cancers)) OR (Advanced Stomach Cancer)) OR (Advanced Gastric Cancers)) OR (Advanced Gastric Cancer))
#3	#1 OR #2
#2	Search: (((Tyrosine Kinase Inhibitors) OR (Tyrosine Kinase Inhibitor)) OR (TKI Tyrosine Kinase Inhibitors)) OR (Tyrosine Protein Kinase Inhibitors)
#1	Search: "Tyrosine Kinase Inhibitors"[Mesh] Sort by: Most Recent

### 2.2 Inclusion and exclusion criteria

The inclusion criteria were as follows: (1) Patients must have a confirmed cytological or pathological diagnosis of clinically advanced GC; (2) RCTs comparing TKIs with non-TKIs were considered eligible; (3) The trials reported one or more of the following outcomes: overall response rate (ORR) (the sum of complete [CR] and partial responses [PR]), DCR (the sum of CR, PR and stable disease [SD]), PFS, OS.

For the purpose of this study, a Partial Response (PR) is defined according to the Response Evaluation Criteria in Solid Tumors (RECIST) guidelines, which specifies that a PR is a ≥30% decrease in the sum of the longest diameters of target lesions, taking as reference the baseline sum diameters. PR must be confirmed by repeat assessment not less than 4 weeks after the criteria for PR are first met, and the subsequent measurements confirm the response.

The exclusion criteria were as follows: (1) duplicate literatures, letters, reviews, editorials, and conference abstracts; (2) inadequate data for outcome estimation; (3) absence of randomized studies; (4) The intervention group did not involve a trial with TKIs.

### 2.3 Data extraction

Two independent investigators (Li and Xue) conducted a comprehensive review of study titles and abstracts. After removing any duplicate publications, full texts were obtained and evaluated based on the following eligibility criteria. In cases where there was disagreement between the two investigators, a third investigator participated in discussion to reach consensus. The collected information from these trials included details such as the first author's name, publication year, study design, patient count, median age, intervention methods employed, clinical trial characteristics including primary endpoint assessment and trial phase classification along with relevant biochemical drugs used. Clinical outcomes collected from the trials included median PFS and OS, HR for both PFS and OS along with their corresponding 95 % CIs, DCR and ORR, RR for DCR and ORR alongside their respective 95 % CIs. ORR was either directly reported or calculated using CRR and PRR. Similarly, DCR was either directly reported or calculated using CRR, PRR and SDR. (ORR was defined as CR plus PR and DCR was defined as ORR plus SD).

### 2.4 Quality assessment

A systematic evaluation of bias in the included trials was performed using the Cochrane criteria. The entries used for the assessment of each study were as follows: random sequence generation, allocation concealment, blinding of participants and personnel, blinding of outcome assessment, incomplete outcome data, selective reporting and other potential sources of bias. The risk level for each domain was categorized as high risk, unclear risk, or low risk based on the degree of alignment between extracted information and evaluation criteria.

In addition to the cochrane risk of bias assessment, we evaluated the overall quality of evidence for each outcome using the GRADE framework, which considers risk of bias, inconsistency, indirectness, imprecision, and publication bias. The evaluation results are shown in **Table 2**.

**Table 2:** Summary of findings for tyrosine kinase inhibitors in advanced gastric cancer.

Outcomes	Assumed risk (Control)	Corresponding risk (TKIs)	Relative effect (95% CI)	Participants (Studies)	Quality of evidence (Grade)	Comments
OS	945 per 1000	840 per 1000	HR 0.76 (0.63-0.92)	1887 (11 RCTs)	⊕⊕⊕◯ Moderate¹	Blinding unclear
PFS	953 per 1000	790 per 1000	HR 0.51 (0.35-0.73)	1764 (10 RCTs)	⊕⊕◯◯ Low²	High heterogeneity
ORR	200 per 1000	406 per 1000	RR 2.03 (0.83-5.01)	1287 (7 RCTs)	⊕⊕◯◯ Low³	Central review missing
DCR	400 per 1000	800 per 1000	RR 3.98 (2.08-7.58)	1510 (7 RCTs)	⊕⊕⊕◯ Moderate^4^	SD assessment variability

¹Downgraded due to unclear blinding.

²Downgraded due to high heterogeneity (I²=91%).

³Downgraded due to unclear central review.

^4^Downgraded due to variability in SD assessment.

### 2.5 Statistic analysis

Meta-analyses were performed using Review Manager (RevMan) version 5.3 and STATA version 12.0. For survival outcomes (OS and PFS), we pooled hazard ratios (HRs) and their 95% confidence intervals (CIs) using the generic inverse-variance method. For dichotomous outcomes (ORR, DCR, adverse events), we calculated risk ratios (RRs) with 95% CIs.

Heterogeneity was assessed using the Cochran's Q test (significance threshold P < 0.10) and quantified with the I² statistic. I² values of 25%, 50%, and 75% were considered to represent low, moderate, and substantial heterogeneity, respectively. Given anticipated clinical and methodological heterogeneity, we employed random-effects models (DerSimonian and Laird method) for all primary analyses. Fixed-effect models were used for sensitivity analyses where appropriate.

### 2.6 Rationale for analyses

The primary analysis aimed to estimate the overall treatment effect of TKI-containing regimens versus non-TKI regimens on OS and PFS … Secondary analyses were conducted to explore potential sources of heterogeneity and assess the consistency of treatment effects across clinically relevant subgroups: (i) by treatment strategy (TKI monotherapy vs TKI plus chemotherapy)…; (ii) by trial phase (II vs III)…; and (iii) by individual TKI agent where sufficient studies existed.

## 3 Results

### 3.1 Study selection

**Figure 1:**
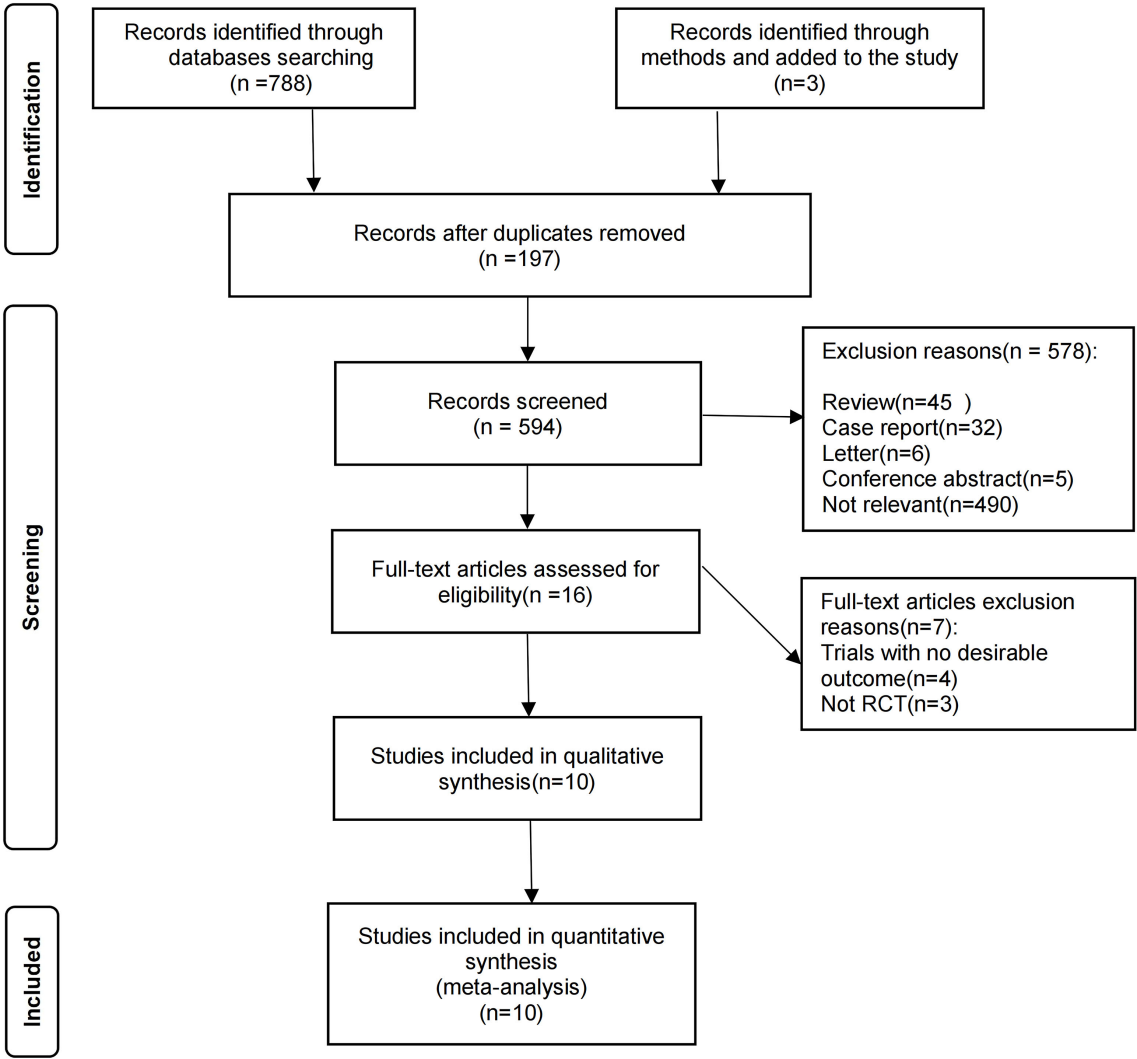
Study selection process.

A total of 788 articles were retrieved from PubMed, Web of Science, and EMBASE databases, and an additional 3 articles were obtained through manual search. Out of these, 197 studies were excluded as duplicates. Following the screening process based on inclusion and exclusion criteria applied to the title, abstract, and keywords of each study, a further 578 studies were excluded. Subsequently, detailed reviews were conducted on the full texts of 16 articles. After removing retrospective articles and reviews that did not meet the inclusion criteria or lacked necessary data, a final selection of 10 studies (4, 9, 19–26) was included in our meta-analysis. The study selection process is illustrated in **Figure 1**.

### 3.2 Study characteristics

The characteristics of the included studies are summarized in **Table 2**, which were published between 2012 and 2024. A total of 1810 patients were enrolled in ten studies, with 1169 patients receiving TKIs or TKIs combined with chemotherapy as intervention and 641 patients receiving placebo or chemotherapy as control. Among these studies, six intervention groups received TKIs alone (4, 9, 19, 22, 23, 25) while four studies (20, 21, 24, 26) received TKIs combined with chemotherapy (TKIs include pazopanib, sunitinib, apatinib, AZD4547, rivoceranib, regorafenib). The control group was treated with placebo or chemotherapy alone or in combination. One study (4) investigated two schedules of apatinib treatment. Additionally, there were 6 phase II studies (4, 21–24, 26) and 4 phase III trials (9, 19, 20, 25) among the included studies.

### 3.3 Assessment of methodological quality

**Figure 2:**
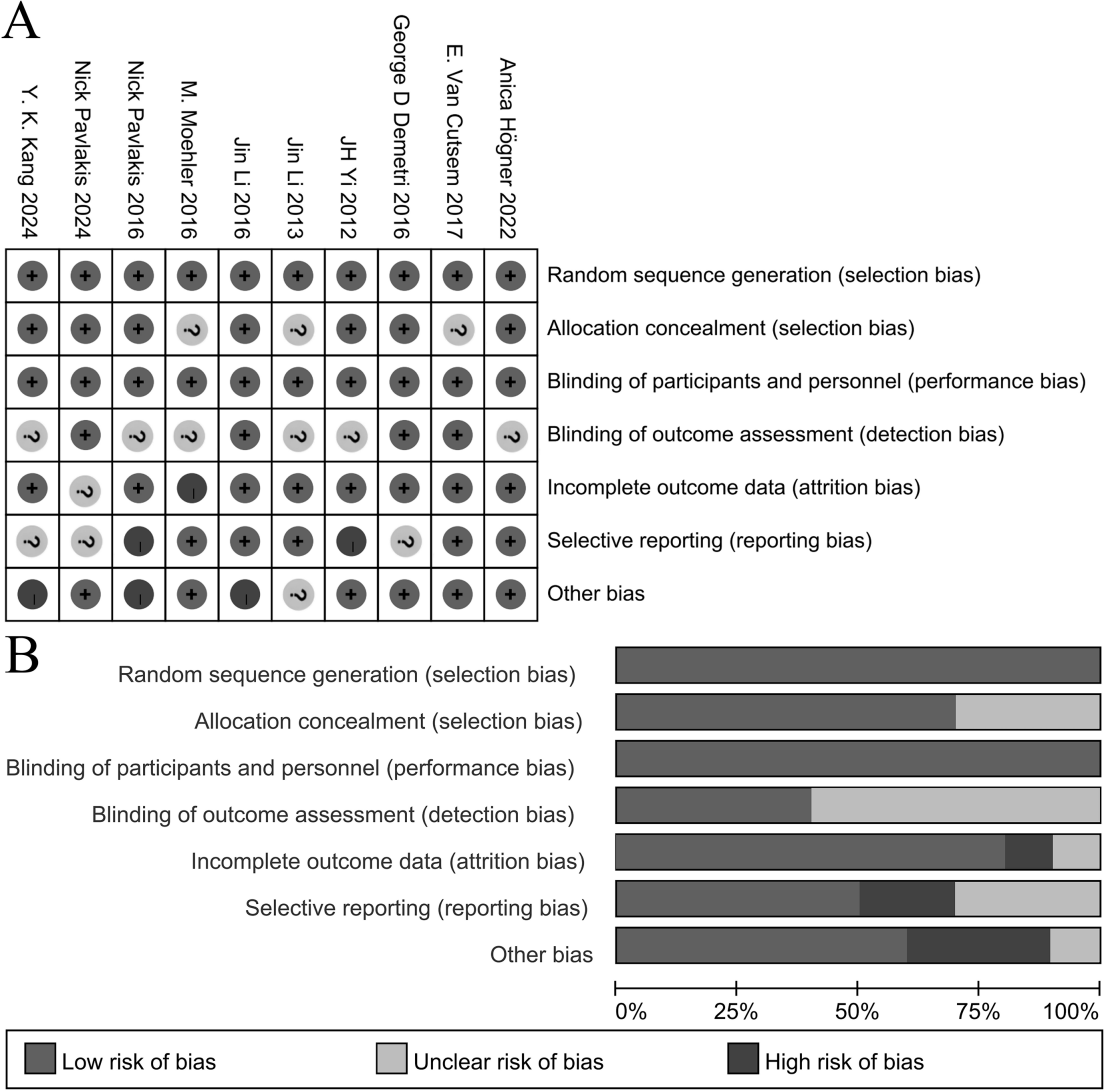
Assessment of risk of bias. **(A)** Risk of bias summary. **(B)** Risk of bias graph.

We critically assessed the methodological quality of the included studies in accordance with the Cochrane Collaboration Risk of Bias Tool. All 10 trials reported adequate randomization, and none of them were terminated prematurely; moreover, all trials were conducted at multiple centers. Consequently, we assigned a low risk of bias rating to these 10 studies regarding randomization. No other sources of bias were identified. The graphical representation depicting the methodological quality is presented in **Figure 2**.

### 3.4 Overall survival and progression-free survival

**Figure 3:**
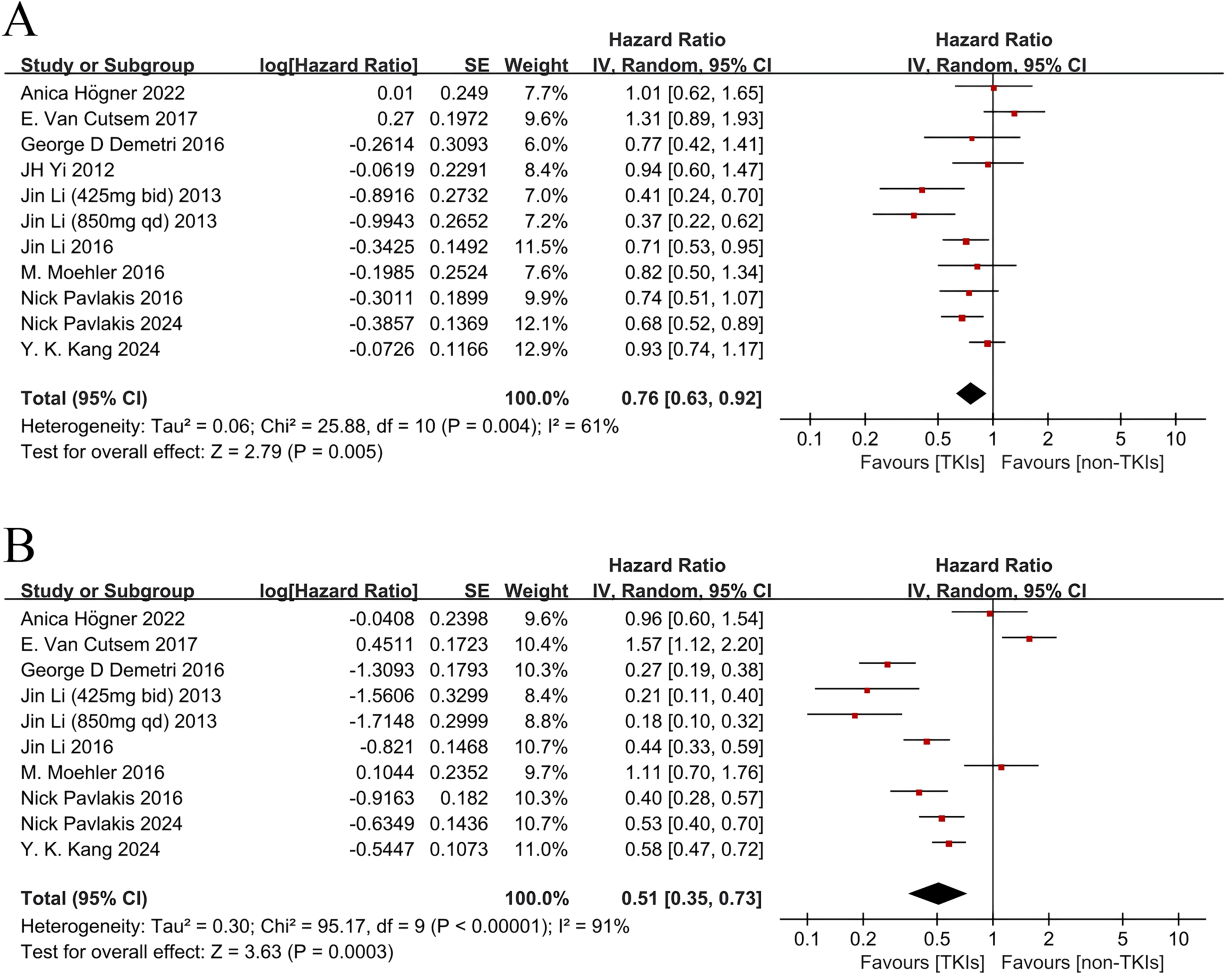
Forest plot and pooled HR and 95 % CI for OS **(A)** and PFS **(B)** tyrosine kinase inhibitors therapy versus non-tyrosine kinase inhibitors therapy. The pooled HR for OS and PFS showed that the patients receiving tyrosine kinase inhibitors therapy possessed a significant improvement in OS and PFS. HR, hazard ratios; OS, overall survival; PFS, progression-free survival; CI, confidence intervals.

The characteristics of patients in analyzed trials are summarized in **Table 3**, All included studies (4, 9, 19–26) reported OS, and nine studies (4, 9, 19–25) reported PFS. Of the ten studies, eight (4, 9, 19–21, 23, 24, 26) reported a statistically significant improvement in OS and nine studies (4, 9, 19–21, 23–26) showed improved PFS. The median OS in the experimental arm ranged from 4.27 to 10.4 months across the included trials, and the median PFS ranged from 1.8 to 4.8 months. A random effect analysis of pooled results revealed that compared to the control arm, treatment with TKIs significantly improved PFS (HR 0.51, 95% CI 0.35-0.73, P = 0.0003, **Figure 3B**) and prolonged OS (HR 0.76, 95% CI 0.63-0.92, P = 0.005, **Figure 3A**), respectively. Significant heterogeneity was detected among the studies in **Figure 3A** (P = 0.004, I^2^ = 61 %) and **Figure 3B** (P < 0.00001, I^2^ = 91 %), prompting us to conduct a sensitivity analysis by excluding certain studies based on their relative weights in each figure's analysis group. Y.K.Kang 's study with the largest relative weight (about 12.9%) was excluded from **Figure 3A** analysis group; George D Demetri 's study with the smallest relative weight (about 6.0%) was also excluded from **Figure 3A** analysis group; Y.K.Kang 's study with the largest relative weight (about 11.0%) was excluded from **Figure 3B** analysis group. It is worth noting that Jin Li's study at a dosage of 425mg (2013), which had the smallest relative weight (about 8.4%), exhibited similar survival outcomes as shown in **Figure 3B**.

**Table 3:** Characteristics of the included studies.

Study	Phase	Line	Treatment arms	No of patients	Median age(year)	Sex (male%)	mOS(m)	mPFS(m)	DCR(%)	ORR(%)
Anica Högner (2022) (21)	II	2	PaFLO	51	65	72	10.19	4.66	72	25
			FLO	27	60	63	7.33	4.47	59	26
JH Yi (2012) (26)	II	2	Docetaxel + sunitinib	56	54	71.4	8	3.9	75	41.4
			Docetaxel	49	52	67.3	6.6	2.6	51	14.3
Jin Li (2016) (9)	III	>2	Apatinib	176	58	75	6.5	2.6	42.05	2.84
			placebo	91	58	75.8	4.7	1.8	8.75	0
E.Van.Cutsem (2017) (22)	II	2	AZD4547	41	51	74	5.5	1.8	NA	2.6
			paclitaxel	30	56	76	6.6	3.5	NA	23.3
Y.K.Kang (2024) (20)	III	2	Rivoceranib + BSC	308	60	78.2	5.78	2.83	40.2	6.5
			placebo + BSC	152	61	73.7	5.13	1.77	13.2	1.3
M.Moehler (2016) (24)	II	2	Sunitinib + FOLFIRI	45	62	73	10.4	3.5	60	20
			placebo + FOLFIRI	46	57	67	8.9	3.3	56	29
Jin Li (2013) (4)	II	>2	Apatinib(850mg qd)	47	55	83	4.83	3.67	51.06	6.38
			Apatinib(425mg bid)	46	53	74	4.27	3.2	34.78	13.04
			placebo	48	54	75	2.5	1.4	10.42	0
George D (2016)	III	2	Regorafenib	133	60	63.9	NA	4.8	52.6	4.5
			placebo	66	61	63.6	NA	0.9	9.1	1.5
Nick Pavlakis (2016) (23)	II	2	Regorafenib	97	63	80	5.8	2.6	NA	NA
			placebo	50	62	80	4.5	0.9	NA	NA
Nick Pavlakis (2024) (19)	III	2	Regorafenib	169	63	72	4.5	1.8	21.3	2.4
			placebo	82	64	84	4	1.6	2.4	0

PaFLO, pazopanib plus 5-fluorouracil, folinic acid, oxaliplatin; FLO, 5-fluorouracil, folinic acid, oxaliplatin; FOLFIRI, 5-fluorouracil, leucovorin and irinotecan; AZD4547, A small molecule tyrosine kinase inhibitor; BSC, best supportive care; mOS, median overall survival; mPFS, median progression-free survival; DCR, disease control rate; ORR, objective response rate; NA, not available.

**Figure 4:**
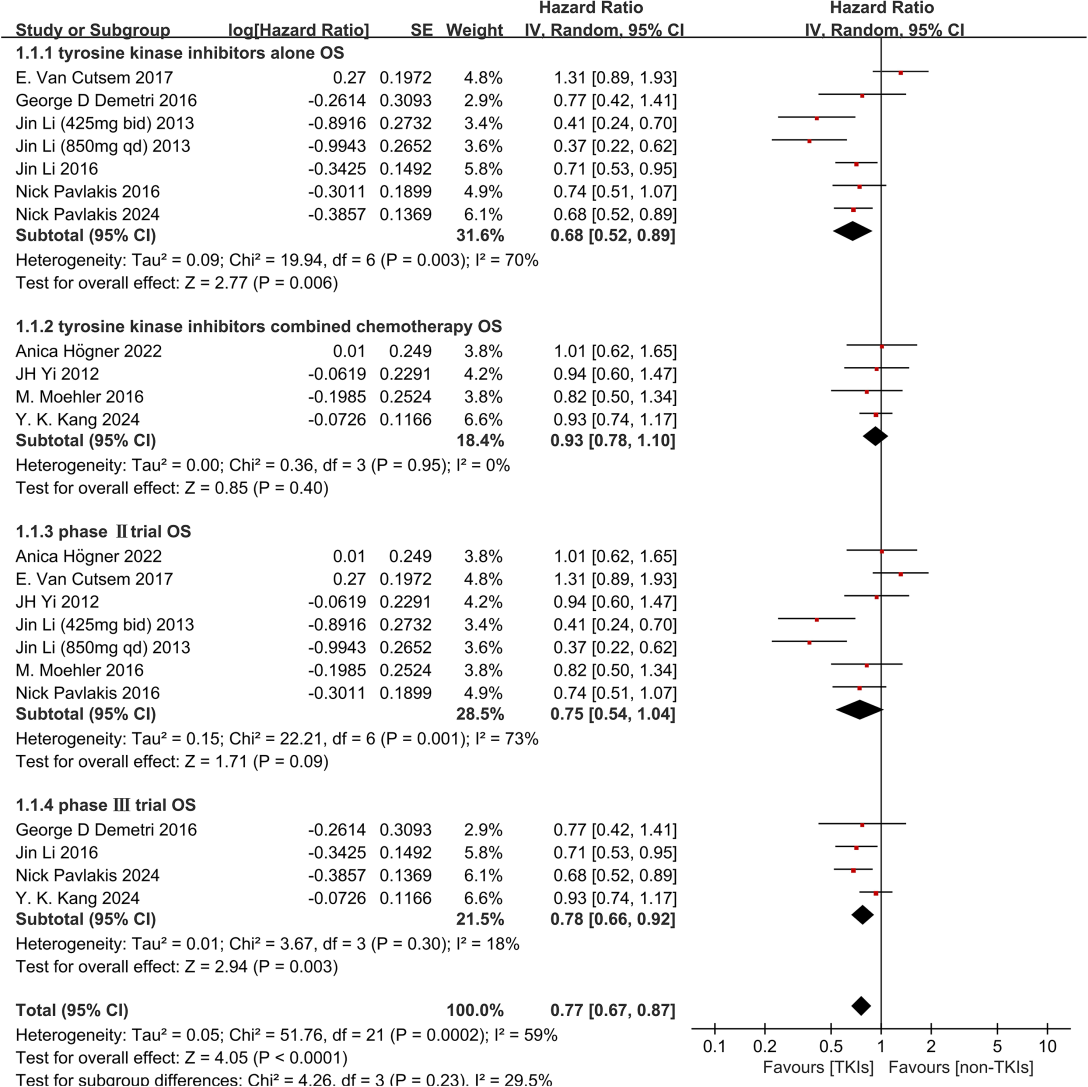
Forest plot and pooled HR and 95% CI for subgroup OS: tyrosine kinase inhibitors therapy versus non-tyrosine kinase inhibitors therapy. HR hazard ratios, CI confidence intervals, OS overall survival. (1.1: OS of subgroups of tyrosine kinase inhibitors alone threapy; 1.2: OS of subgroups of tyrosine kinase inhibitors combined with chemo-therapy; 1.3: OS of subgroups of the phase II trial; 1.4: OS of subgroups of the phase III trial).

**Figure 5:**
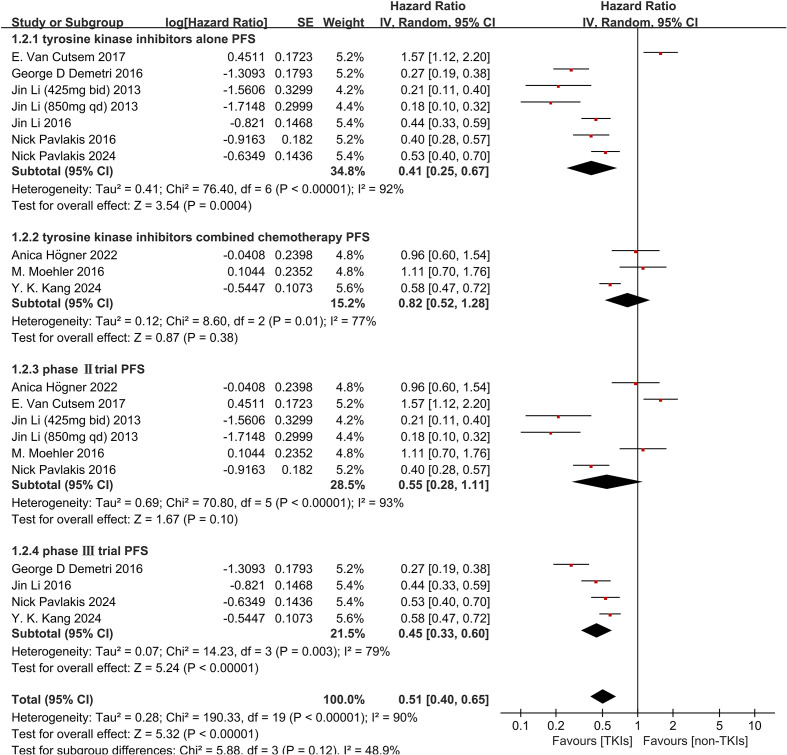
Forest plot and pooled HR and 95% CI for subgroup PFS: tyrosine kinase inhibitors therapy versus non-tyrosine kinase inhibitors therapy. HR hazard ratios, CI confidence intervals, PFS progression-free survival. (2.1: PFS of subgroups of tyrosine kinase inhibitors alone threapy; 2.2: PFS of subgroups of tyrosine kinase inhibitors combined with chemo-therapy; 2.3: PFS of subgroups of the phaseIItrial; 2.4: PFS of subgroups of the phase III trial).

In the subgroup analysis of treatment administration, monotherapy with TKIs alone demon-strated a significant improvement in OS (HR 0.68, 95%CI 0.52-0.89, P = 0.006, **Figure 4**) and significantly prolonged PFS (HR 0.41, 95% CI 0.25-0.67, *P* = 0.0004, **Figure 5**), However, the combination of chemotherapy and TKIs did not show a significant benefit for OS (HR 0.93, 95% CI 0.78-1.10, P = 0.40, **Figure 4**) or PFS (HR 0.82, 95% CI 0.52-1.28, P = 0.38, **Figure 5**), compared to chemotherapy alone. In the Phase III subgroup, the results showed significant improvements in both OS (HR 0.78, 95% CI 0.66-0.92, P = 0.003, **Figure 4**) and PFS (HR 0.45, 95% CI 0.33-0.60, P < 0.0001, **Figure 5**). In addition, the Phase II trials all exhibited a tendency towards improved OS (HR 0.75, 95% CI 0.54-1.04, P = 0.09, **Figure 4**) and PFS (HR 0.55, 95% CI 0.28-1.11, P = 0.10, **Figure 5**). However, a moderate heterogeneity was observed among the Phase II clinical trials subgroups.

Therefore, in order to obtain comparable pooled estimates, a random effects model was employed for all aforementioned analyses.

### 3.5 Overall response rate and disease control rate

**Figure 6:**
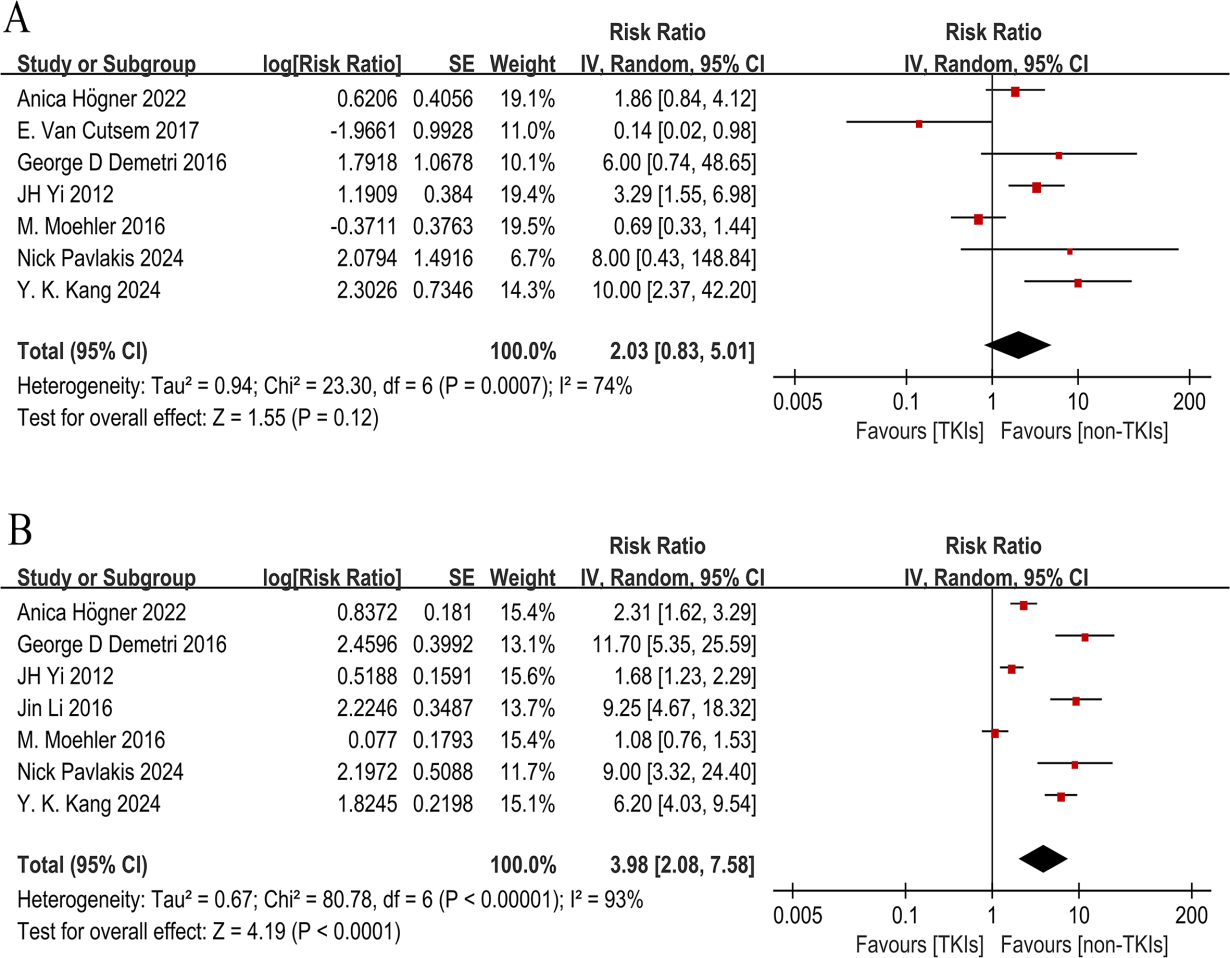
Forest plot and pooled RR and 95 % CI for ORR **(A)** and DCR **(B)**: tyrosine kinase inhibitors therapy versus non-tyrosine kinase inhibitors therapy. The pooled RR for DCR and ORR showed that the patients receiving tyrosine kinase inhibitors therapy possessed a significant improvement in DCR, however, no significant benefit was found for ORR. RR risk ratios, CI confidence intervals, ORR objective response rate, DCR disease control rate.

A total of seven trials reported ORR, while seven studies reported DCR. The DCR range in the TKI group was 21.3% to 75%, and the ORR range was 2.4% to 41.4%. Pooled data demonstrated that TKIs exhibited a higher ORR (RR 2.03, 95% CI 0.83-5.01, P = 0.12, **Figure 6A**) and a higher DCR (RR 3.98, 95% CI 2.08-7.58, P = 0.0001, **Figure 6B**) than non-TKIs. However, due to significant heterogeneity with other experimental groups, the study by Jin Li (2013) was excluded from the forest plot analysis. Among all experimental groups, JH Yi (2012) reported the highest ORR (41.4%) and DCR (75%).

**Figure 7:**
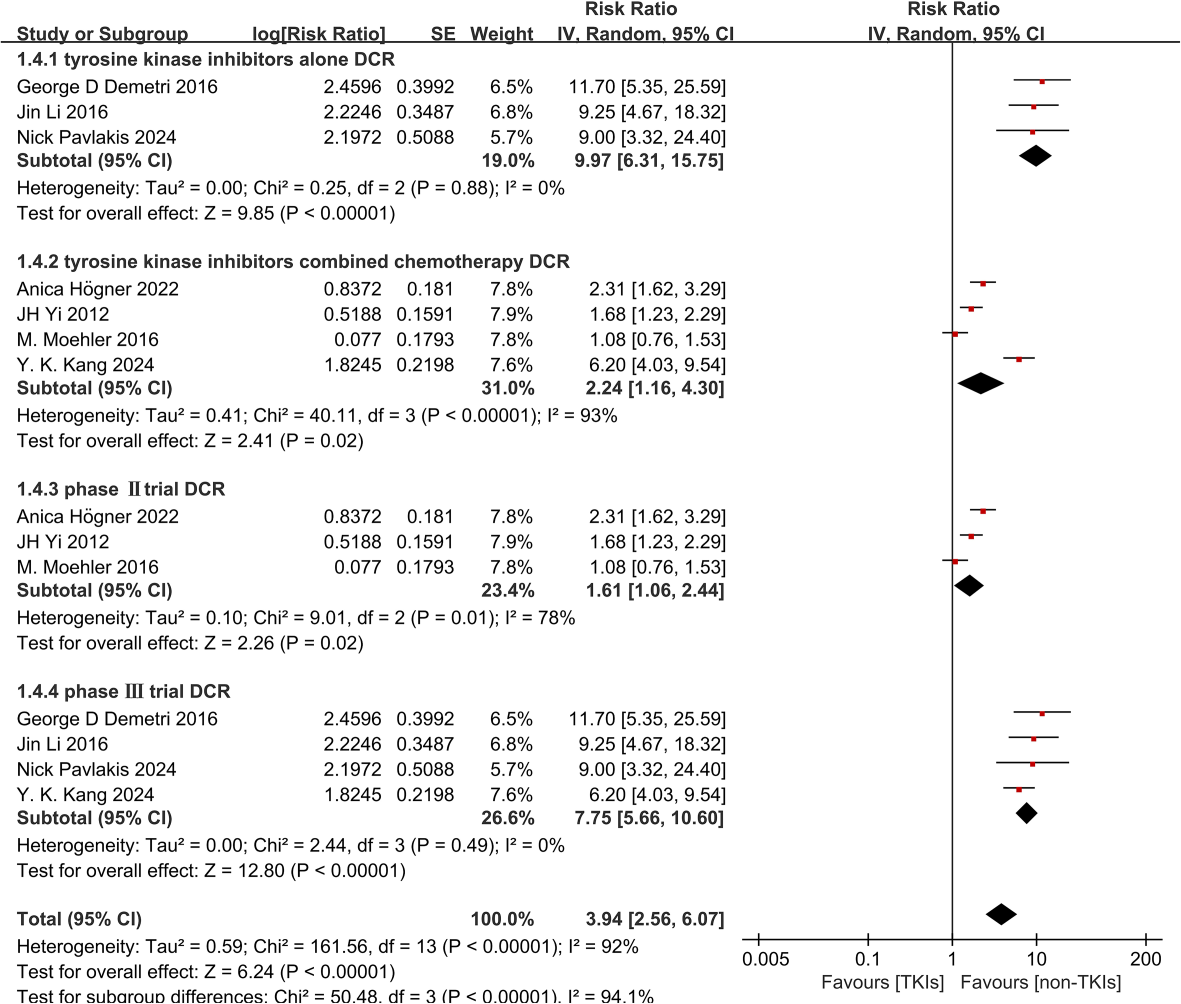
Forest plot and pooled RR and 95% CI for subgroup DCR: tyrosine kinase inhibitors therapy versus non-tyrosine kinase inhibitors therapy. RR risk ratios, CI confidence intervals, DCR disease control rate. (4.1: DCR of subgroups of tyrosine kinase inhibitors alone threapy; 4.2: DCR of subgroups of tyrosine kinase inhibitors combined with chemo-therapy; 4.3: DCR of subgroups of the phaseIItrial; 4.4: DCR of subgroups of the phaseIIItrial).

**Figure 8:**
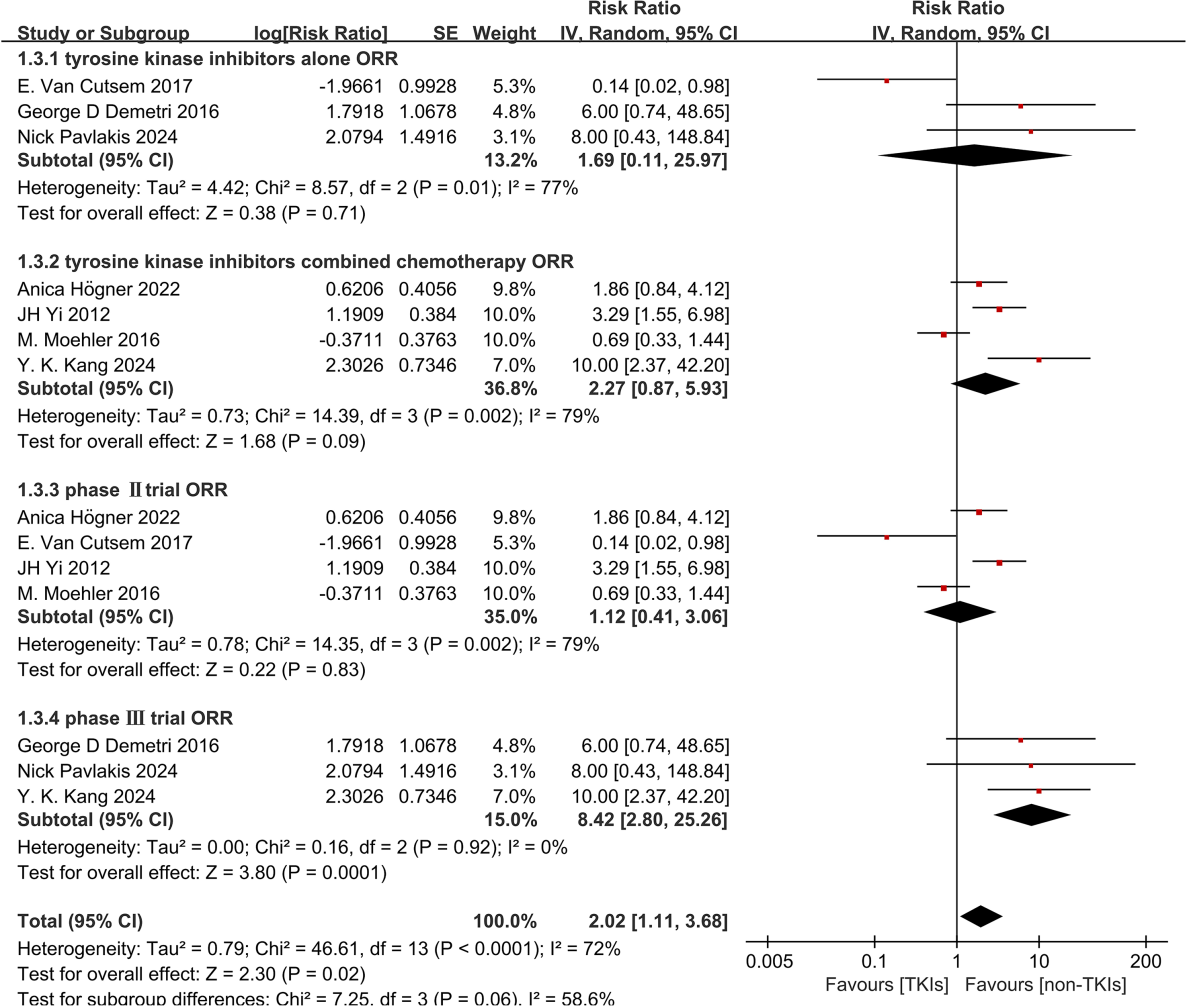
Forest plot and pooled RR and 95% CI for subgroup ORR: tyrosine kinase inhibitors therapy versus non-tyrosine kinase inhibitors therapy. RR risk ratios, CI confidence intervals, ORR objective response rate. (3.1: ORR of subgroups of tyrosine kinase inhibitors alone threapy; 3.2: ORR of subgroups of tyrosine kinase inhibitors combined with chemo-therapy; 3.3: ORR of subgroups of the phaseIItrial; 3.4: ORR of subgroups of the phaseIII trial).

Subgroup analysis revealed that both TKIs monotherapy and combination therapy with chemotherapy significantly improved DCR (RR 9.97, 95% CI 6.31-15.75, P < 0.0001, **Figure 7** and RR 2.24, 95% CI 1.16-4.30, P = 0.02, **Figure 7**). However, there was no observed improvement in ORR with either TKIs alone or in combination with chemotherapy (RR 1.69, 95% CI 0.11-25.97, P = 0.71, **Figure 8** and RR 2.27, 95% CI 0.87-5.93, P = 0.09, **Figure 8**). Due to significant heterogeneity within both groups, we used the random effects model for analysis. Furthermore, treatment with TKIs demonstrated a significant enhancement in DCR across Phase II and Phase III trials (RR 1.61, 95% CI 1.06-2.44, P = 0.02, **Figure 7** and RR 7.75, 95% CI 5.66-10.60, P < 0.0001, **Figure 7**). In Phase III clinical trials, the use of TKIs resulted in a notable improvement in ORR (RR 8.42, 95% CI 2.80-25.26, P = 0.0001, **Figure 8**). Although there was a tendency towards an increased ORR (RR 1.12, 95% CI 0.41-3.06, P = 0.83, **Figure 8**) in Phase II trials, the high level of heterogeneity rendered it statistically insignificant.

### 3.6 Safety

The toxicities reported in the included studies are presented in **Table 3** (only grade ≥3 toxicities are shown). Overall, in addition to the common toxicities of chemotherapy, the incidence of specific toxicities associated with TKIs may reflect their safety profile. These include anemia, hypertension, leukopenia, diarrhea, neutropenia, abdominal pain, hand-foot syndrome, nausea, vomiting, fatigue, proteinuria, thromboembolic events, thrombocytopenia, decreased appetite, and bleeding. Notably, patients treated with TKIs had significantly higher frequencies of hypertension (RR 31.5 95% CI 11.71-84.73, P < 0.001, **Table 4**) and hand-foot syndrome (RR 55, 95% CI 7.65-395.32, P = 0.0001, **Table 4**). Furthermore, patients treated with TKIs exhibited higher incidences of leucopenia (RR 2, 95% CI 1.12-3.57, P = 0.019, **Table 3**), diarrhea (RR 3.36, 95% CI 1.73-6.54, P = 0.0017, **Table 4**), neutropenia (RR 2.96, 95% CI 1.89-4.62, P < 0.001, **Table 4**), nausea (RR 2.38, 95% CI 1.05-9.39, P = 0.015, **Table 4**), fatigue (RR 2.56, 95% CI 1.50-4.36, P = 0.0076, **Table 4**), thrombocytopenia (RR 2.8, 95% CI 1.02-7.67, P = 0.045, **Table 4**), and decreased appetite (RR 3.2, 95% CI 1.60-6.41, P = 0.001, **Table 4**). The relative risks (RRs) of grade ≥ 3 adverse events are summarized in **Table 4**.

**Table 4:** RR of grade ≥3 adverse events in patients with advanced gastric cancer.

Grade ≥3 adverse events	No.of trials	Events/total		RR (95 % CI)	P value	Analysis model
		Treatment group	Control group			
Anaemia	6	67/852	45/448	1.49(1.04-2.13)	0.009	Fixed
Hypertension	6	126/975	4/488	31.5(11.71-84.73)	0.001	Fixed
Leucopenia	5	32/421	16/260	2(1.12-3.57)	0.019	Fixed
Diarrhea	8	37/1030	11/560	3.36(1.73-6.54)	0.0017	Fixed
Neutropenia	5	68/421	23/260	2.96(1.89-4.62)	0.001	Fixed
Abdominal pain	5	11/266	17/376	0.65(0.31-1.36)	0.057	Fixed
Hand-foot syndrome	5	55/509	1/281	55(7.65-395.32)	0.0001	Fixed
Nausea	7	19/854	8/468	2.38(1.05-9.39)	0.015	Fixed
Vomiting	5	9/414	8/251	1.13(0.44-2.88)	0.761	Fixed
Fatigue	7	46/974	18/510	2.56(1.50-4.36)	0.0076	Fixed
Proteinuria	3	3/93	1/290	3(0.316-28.50)	0.3387	Fixed
Thromboembolic events	1	2/45	1/45	2(0.19-21.28)	0.5638	Fixed
Thrombocytopenia	4	14/376	5/215	2.8(1.02-7.67)	0.045	Fixed
Decreased appetite	3	32/534	10/269	3.2(1.60-6.41)	0.001	Fixed
Bleeding or Hemorrhage	2	16/345	13/173	1.23(0.61-2.50)	0.7789	Fixed

RR, risk ratios; Fixed, Fixed effect model.

### 3.7 Sensitivity analysis and publication bias

**Figure 9:**
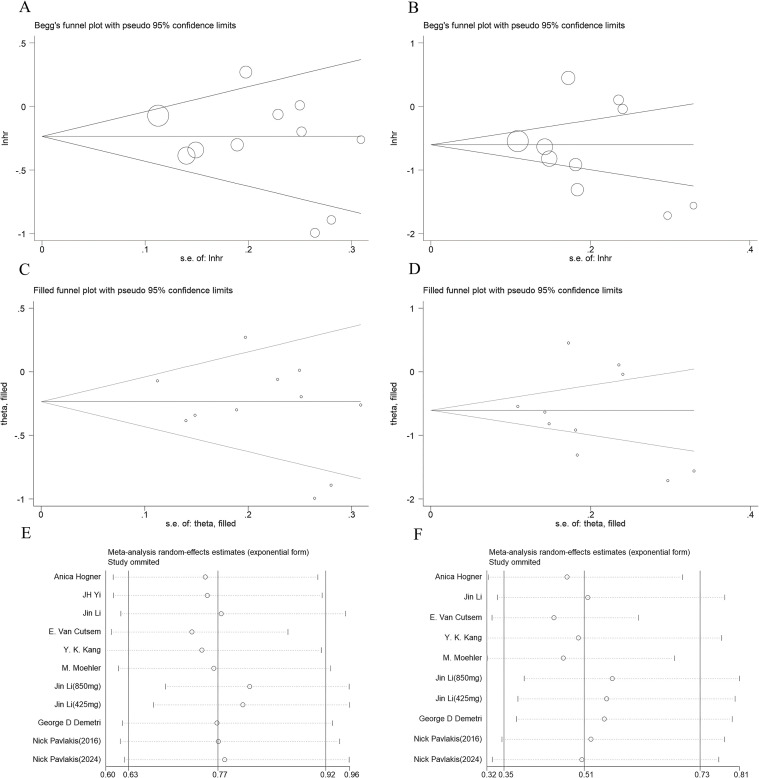
**(A)** Begg’s funnel plot analysis and **(C)** trimming and filling analysis for evaluating publication bias for OS; and **(B)** Begg’s funnel plot analysis and **(D)** trimming and filling analysis for evaluating publication bias for PFS. Sensitivity analyses were conducted to clarify the impact of individual studies on the **(E)** OS and **(F)** PFS results. Begg’s funnel plots show no significant publication bias among the studies included here. The results for OS and PFS remained stable after trimming and filling analysis. The sensitivity analyses show the robustness of the results. lnhr, log of hazard ratio; s.e. standard error.

Sensitivity analyses were performed using Stata 12.0 to assess the impact of individual studies on the overall results, with primary indicators being OS and PFS, and secondary indicators being ORR and DCR. The exclusion of any single study did not significantly alter the overall HR estimates for OS (**Figure 9E**) or PFS (**Figure 9F**), thus confirming the robustness of our findings.

To evaluate potential publication bias, Begg’s funnel plots and Egger’s tests were employed. No significant publication bias was detected among the studies using Begg’s funnel plots (**Figures 9A, B**). Additionally, these findings were corroborated using Egger’s test for OS (P = 0.397) and PFS (P = 0.592). After conducting trimming and filling analyses, stable results were obtained for OS (HR = 0.765, 95% CI = 0.634-0.924) (**Figure 9C**) and PFS (HR = 0.506, 95% CI = 0.332-0.770) (**Figure 9D**).

## 4 Discussion

This updated systematic review and meta-analysis provides a comprehensive synthesis of efficacy and safety outcomes for TKI-containing regimens in advanced gastric cancer, incorporating the most recent evidence through March 2026. Our primary findings demonstrate that TKIs significantly improve overall survival (HR 0.76, 95% CI 0.63-0.92; moderate certainty), progression-free survival (HR 0.51, 95% CI 0.35-0.73; low certainty), and disease control rate (RR 3.98, 95% CI 2.08-7.58; moderate certainty) compared to non-TKI regimens. The objective response rate showed a non-significant trend favoring TKIs (RR 2.03, 95% CI 0.83-5.01; low certainty), with substantial heterogeneity across studies. Critically, subgroup analyses revealed that the survival benefit was confined to TKI monotherapy, whereas the addition of TKIs to chemotherapy did not confer significant improvement over chemotherapy alone. This differential effect has important implications for clinical trial design and treatment selection. The safety profile was characterized by predictable, mechanism-based toxicities, most notably hypertension and hand-foot syndrome, which were manageable with appropriate monitoring and supportive care.

Our findings extend and refine those of prior meta-analyses (12–18) in several important respects. First, by incorporating recently completed phase III trials (19, 20, 25), we provide updated estimates with improved precision. Second, the rigorous subgroup analysis by treatment strategy clarifies a previously ambiguous area: while individual trials of TKI-chemotherapy combinations yielded mixed results (20, 21, 24, 26), our pooled analysis suggests that combination therapy does not provide additive survival benefit. This finding aligns with the mechanistic consideration that overlapping toxicities may compromise dose intensity, and that adding TKIs to multi-agent chemotherapy may not be the optimal strategy for leveraging their anti-angiogenic effects. The substantial PFS benefit (HR 0.51) contrasts with the more modest OS benefit (HR 0.76), a pattern commonly observed in targeted therapy trials where post-progression treatments may dilute survival differences. This discrepancy underscores the importance of considering both endpoints in treatment decisions and highlights the need for careful attention to subsequent-line therapies in trial design. Our findings regarding differential efficacy by treatment line and TKI agent are constrained by the limited number of studies available for these subgroup analyses. However, the consistency of benefit across phase III trials supports the generalizability of our conclusions, while the non-significant trends in phase II trials likely reflect limited statistical power rather than true absence of effect.

The observed efficacy of TKI monotherapy, particularly in later lines of treatment, can be understood through the mechanism of VEGF pathway inhibition in gastric cancer. Tumor angiogenesis, driven by VEGF signaling, is a critical process in gastric cancer progression and metastasis (27–30). TKIs such as apatinib, regorafenib, and rivoceranib exert their antitumor effects by selectively binding to the ATP-binding site of VEGFR tyrosine kinases, disrupting downstream signal transduction and inhibiting endothelial cell proliferation and migration (31–33). This anti-angiogenic effect reduces tumor blood supply, leading to growth inhibition and, in some cases, tumor regression. The lack of additive benefit when TKIs are combined with chemotherapy may reflect several factors. First, chemotherapy-induced cytotoxicity can damage endothelial cells, potentially counteracting the anti-angiogenic effects of TKIs. Second, overlapping toxicities may necessitate dose reductions of either agent, compromising efficacy. Third, the patient populations enrolled in combination trials may have had poorer prognosis or higher burden of disease, limiting the ability to detect incremental benefit. Finally, the optimal sequencing of TKIs and chemotherapy—whether concurrent or sequential—remains undefined and warrants prospective investigation.

The safety profile observed in this analysis is consistent with the known class effects of anti-angiogenic TKIs. Hypertension, the most frequently observed grade ≥3 adverse event (RR 31.5), results from VEGFR inhibition in vascular endothelium, leading to reduced nitric oxide production, vasoconstriction, and increased peripheral vascular resistance (34–36). Hand-foot syndrome (RR 55.0) reflects local accumulation of TKIs in capillaries of palms and soles, exacerbated by mechanical stress and friction. The hematologic toxicities—neutropenia, leukopenia, thrombocytopenia, and anemia—are attributable to off-target inhibition of kinases involved in hematopoietic cell survival and proliferation (35, 36). Importantly, while these toxicities occur at significantly higher rates with TKIs, they are largely predictable and manageable with appropriate monitoring, patient education, and supportive care interventions. Dose modifications, treatment interruptions, and antihypertensive therapy can effectively mitigate these effects in most patients, supporting the feasibility of TKI use in clinical practice (31).

This meta-analysis has several methodological strengths. We conducted an updated search through March 2026, capturing the most recent trial evidence and ensuring contemporary relevance. We adhered strictly to PRISMA 2020 guidelines and prospectively registered the protocol, enhancing transparency and reproducibility. We employed rigorous quality assessment using the Cochrane RoB 2 tool and GRADE framework, providing transparent certainty ratings for each outcome. We performed comprehensive subgroup analyses to explore heterogeneity and identify effect modifiers, yielding clinically actionable insights. Sensitivity analyses confirmed the robustness of our findings, and formal publication bias assessment suggested minimal risk of selective reporting. The inclusion of detailed safety data provides a balanced assessment of the risk-benefit profile.

Several limitations warrant consideration when interpreting our findings. First, the number of included studies was relatively modest (10 RCTs), limiting statistical power for some subgroup analyses and precluding meta-regression to explore multiple sources of heterogeneity simultaneously. Second, substantial heterogeneity was observed for several outcomes, particularly PFS (I²=91%) and ORR (I²=83%). While we explored potential sources through subgroup analyses, residual heterogeneity attributable to differences in patient populations, TKI agents, dosing schedules, and outcome definitions likely remains. Third, our analyses are based on aggregate trial-level data rather than individual patient data, which precludes more nuanced exploration of effect modification by patient characteristics (e.g., age, performance status, biomarker status). Fourth, publication bias assessment using funnel plots and Egger's test has limited sensitivity when fewer than 10 studies are available, though the symmetrical appearance of plots and trim-and-fill analyses support the stability of findings. Fifth, the exclusion of non-English language publications (though not by design) may have introduced language bias, although our comprehensive database search minimized this risk. Sixth, variability in outcome definitions—particularly for stable disease assessment in DCR—may have contributed to heterogeneity and affected GRADE certainty ratings. Finally, the relatively short follow-up in some trials limits assessment of long-term survival outcomes and late-onset toxicities.

From a clinical perspective, our findings support the continued use of TKI monotherapy as a treatment option for patients with advanced gastric cancer, particularly in later lines of therapy where treatment options are limited. The significant improvement in OS and PFS, coupled with a manageable safety profile, positions TKIs as valuable agents in the therapeutic armamentarium. The differential benefit observed with monotherapy suggests that TKIs may be most effectively deployed as single agents following chemotherapy failure, rather than combined with chemotherapy upfront. For patients receiving TKIs, clinicians should implement proactive monitoring for class-specific toxicities. Blood pressure should be assessed at baseline and regularly during treatment, with prompt initiation of antihypertensive therapy if hypertension develops. Dermatologic evaluation and patient education regarding hand-foot syndrome prevention (emollients, avoidance of friction, protective footwear) are essential. Regular blood count monitoring is warranted to detect and manage hematologic toxicities. Dose modifications should be considered according to product labeling and clinical judgment when significant toxicities occur. Importantly, the absence of reliable predictive biomarkers for TKI efficacy in gastric cancer remains a significant challenge (37, 38). Unlike in other tumor types where VEGFR expression or genetic alterations guide patient selection, no validated biomarker currently exists to identify which patients with gastric cancer are most likely to benefit from TKI therapy. This represents a critical knowledge gap and an urgent priority for future research.

For future research, this meta-analysis highlights several priorities. First, the development and validation of predictive biomarkers to enable personalized TKI selection is paramount. Studies should explore whether VEGFR expression levels, circulating angiogenic factors, or tumor genomic alterations (e.g., VEGFR2 amplification, angiogenesis-related gene signatures) predict response to TKI therapy (37, 38). Second, the optimal sequencing of TKIs relative to chemotherapy and immunotherapy requires prospective evaluation. Given our finding that concurrent combination with chemotherapy did not improve outcomes, future trials should investigate sequential strategies—TKI therapy following chemotherapy progression, or intercalated approaches—that may optimize efficacy while minimizing toxicity. Third, dose optimization studies are needed to define the minimal effective dose that balances efficacy and tolerability. The observation by Nick Pavlakis et al. (19, 39, 40) that lower starting doses of regorafenib with gradual dose escalation reduces adverse events without compromising efficacy warrants further investigation across TKI agents. Fourth, combination strategies with immunotherapy merit exploration. Recent evidence suggests that regorafenib plus nivolumab yields favorable antitumor responses with acceptable tolerability in immune-sensitive populations (39–41). The mechanistic rationale—that TKIs may modulate the tumor microenvironment and enhance immune checkpoint inhibitor efficacy—supports continued investigation of such combinations. Fifth, head-to-head comparisons of different TKIs are needed to determine whether efficacy and safety profiles differ meaningfully among agents. While indirect comparisons from our meta-analysis suggest comparable effects, direct randomized comparisons would provide definitive evidence. Finally, future trials should incorporate standardized outcome definitions, centralized response review, and patient-reported outcomes to enhance comparability across studies and provide a more complete assessment of treatment impact.

The therapeutic landscape for advanced gastric cancer is evolving rapidly, with emerging targeted therapies and immunotherapies expanding treatment options. Within this evolving context, TKIs will likely remain important agents, particularly for patients who have progressed on or are ineligible for other treatments. The integration of TKIs into treatment algorithms will increasingly depend on biomarker-driven patient selection and rational combination strategies informed by tumor biology. Molecular profiling of gastric cancer has revealed substantial heterogeneity, with distinct molecular subtypes (e.g., EBV-positive, microsatellite unstable, genomically stable, chromosomally unstable) that may differentially respond to targeted therapies (37, 38). Future research should explore whether TKI efficacy varies by molecular subtype, enabling more precise patient selection. The challenge of acquired resistance to TKIs—mediated by mechanisms including alternative angiogenic pathways, tumor microenvironment adaptations, and epigenetic changes—requires sustained investigation (42). Understanding resistance mechanisms will inform the development of next-generation TKIs and rational combination strategies to overcome or delay resistance.

## 5 Conclusions

This updated systematic review and meta-analysis provides moderate-certainty evidence that TKI-containing regimens significantly improve overall survival, progression-free survival, and disease control rate in patients with advanced gastric cancer. The survival benefit is primarily attributable to TKI monotherapy, whereas combination with chemotherapy does not confer additive benefit. The safety profile is characterized by predictable, mechanism-based toxicities—particularly hypertension and hand-foot syndrome—that are manageable with appropriate monitoring and supportive care.

These findings refine the current evidence base and support the continued role of TKIs in treatment algorithms for advanced gastric cancer, particularly in later lines of therapy. However, critical knowledge gaps remain, including the absence of validated predictive biomarkers, optimal treatment sequencing, and mechanisms of acquired resistance. Addressing these gaps through rigorous translational research and well-designed clinical trials will be essential to maximize the therapeutic potential of TKIs and advance precision oncology in gastric cancer.

## Data Availability

The original contributions presented in the study are included in the article/**Supplementary Material**. Further inquiries can be directed to the corresponding author/s.
