# Supplementary material for: Tyrosine kinase inhibitors and survival in advanced or metastatic gastric cancer: a systematic review and meta-analysis
Source: Front Oncol. 2026 Mar 24;16:1771234. doi: 10.3389/fonc.2026.1771234 (PMC13053319; doi:10.3389/fonc.2026.1771234)
Supplement: Supplementary file 1 [file SupplementaryFile1.docx]

| **Section and Topic** | **Item #** | **Checklist item** | **Location where item is reported** |
| --- | --- | --- | --- |
| **TITLE** | | |  |
| Title | 1 | Effect of tyrosine kinase inhibitors on survival in patients with advanced or metastatic gastric cancer: a systematic review and meta-analysis |  |
| **ABSTRACT** | | |  |
| Abstract | 2 | Background: Small-molecule tyrosine kinase inhibitors (TKIs) have been extensively utilized in the management of advanced or metastatic gastric cancer (GC) across numerous studies; however, their specific efficacy remains a topic of ongoing debate. This study aimed to conduct a comprehensive evaluation of the impact of TKIs on advanced GC through a meta-analysis.  Methods: A comprehensive literature search was performed in PubMed, Web of Science, and Embase databases for articles published up to Oct 20, 2024. Data extraction was performed following a rigorous screening process based on predefined inclusion and exclusion criteria. Hazard ratios (HRs) with corresponding 95% confidence intervals (CIs) were employed to quantify the progression-free survival (PFS) and overall survival (OS) data. Risk ratios (RRs) and 95% CIs were pooled for objective response rate (ORR) and disease control rate (DCR).  Results The meta-analysis incorporated ten randomized controlled trials (RCTs) encompassing a total of 1810 patients, with 1169 patients in the experimental group and 641 patients in the control group. The analysis revealed treatment regimens including TKIs significantly enhanced overall survival (OS) (HR 0.76, 95% CI 0.63-0.92, P = 0.005) and prolonged progression-free survival (PFS) (HR 0.51, 95% CI 0.35-0.73, P = 0.0003) compared to regimens excluding TKIs. Additionally, DCR exhibited a a marked improvement (RR 3.98, 95% CI 2.08-7.58, P ＜ 0.0001), while ORR did not show a statistically significant reduction (RR 2.03, 95% CI 0.83-5.01, P = 0.12). Although TKIs were associated with adverse events such as anemia, diarrhea, hypertension, and hand-foot syndrome, a relative risk assessment of grade ≥ 3 adverse events suggested that these events were largely predictable and manageable.  Conclusions: Regimens containing TKIs demonstrated superior efficacy in terms of OS, PFS, and DCR compared to those without TKIs in patients with advanced GC, with their toxicity being within a controllable range. |  |
| **INTRODUCTION** | | |  |
| Rationale | 3 | Given the inconclusive results from invarious phase I/II trials assessing anti-angiogenic TKIs and the ongoing controversy surrounding their efficacy in advanced or metastatic GC, the overall effectiveness and safety of TKIs in this context remain uncertain. Therefore, further research is warranted to elucidate the role of TKIs in the treatment of advanced or metastatic GC. |  |
| Objectives | 4 | In this study, we conducted an updated meta-analysis to consolidate and evaluate the efficacy and safety data of TKIs in patients with advanced GC. |  |
| **METHODS** | | |  |
| Eligibility criteria | 5 | The inclusion criteria were as follows: (1) Patients must have a confirmed cytological or pathological diagnosis of clinically advanced GC; (2) RCTs comparing TKIs with non-TKIs were considered eligible; (3) The trials reported one or more of the following outcomes: overall response rate (ORR) (the sum of complete [CR] and partial responses [PR]), DCR (the sum of CR, PR and stable disease [SD]), PFS, OS.  The exclusion criteria were as follows: (1) duplicate literatures, letters, reviews, editorials, and conference abstracts; (2) inadequate data for outcome estimation; (3) absence of randomized studies; (4) The intervention group did not involve a trial with TKIs. |  |
| Information sources | 6 | To identify relevant articles, we comprehensively searched PubMed, EMBASE, and Web of Science databases up until October 20, 2024. After eliminating duplicate records, we screened titles and abstracts followed by a thorough evaluation of full texts. Any discrepancies regarding inclusion criteria were resolved through consensus discussion. |  |
| Search strategy | 7 | **PUBMED：**  Search number Search terms  #5 #3 AND #4  #4 Search: (((((((((Advanced Stomach Neoplasms) OR (Advanced Stomach Neoplasm)) OR (Advanced Gastric Neoplasms)) OR (Advanced Gastric Neoplasm)) OR (Advanced Cancer of Stomach)) OR (Advanced Stomach Cancers)) OR (Advanced Stomach Cancer)) OR (Advanced Gastric Cancers)) OR (Advanced Gastric Cancer))  #3 #1 OR #2  #2 Search: (((Tyrosine Kinase Inhibitors) OR (Tyrosine Kinase Inhibitor)) OR (TKI Tyrosine Kinase Inhibitors)) OR (Tyrosine Protein Kinase Inhibitors)  #1 Search: "Tyrosine Kinase Inhibitors"[Mesh] Sort by: Most Recent |  |
| Selection process | 8 | The selection process consisted of three stages: (1) title and abstract screening, (2) full-text review, and (3) final inclusion based on predefined eligibility criteria. Two independent reviewers (Yuhui Xue and Shantong Liu) screened all records retrieved from PubMed, EMBASE, and Web of Science databases. Duplicate records were removed using EndNote software. Any disagreements during the selection process were resolved through consensus discussion or by consulting a third reviewer. The PRISMA flow diagram illustrates the number of studies screened, assessed, and included. |  |
| Data collection process | 9 | Data extraction was performed by three independent evaluators (Y.X., S.L. and Y.Z. They screen eligible studies and extract relevant data into predefined standardized tables. Data included study characteristics (e.g., author, year of publication, sample size, intervention details), outcomes (e.g., progression-free survival, overall survival, objective response rate), and methodological details (e.g., study design, risk of bias). Differences between the evaluators were resolved by consensus or consultation with a third researcher. No automated tools were used in the data extraction process. |  |
| Data items | 10a | The outcomes sought for this meta-analysis included:  Primary outcomes: Overall survival (OS) and progression-free survival (PFS), expressed as hazard ratios (HRs) with 95% confidence intervals (CIs).  Secondary outcomes: Objective response rate (ORR, the sum of complete response and partial response) and disease control rate (DCR, the sum of ORR and stable disease), expressed as risk ratios (RRs) with 95% CIs.  Safety outcomes: The incidence of grade ≥3 adverse events (e.g., anemia, hypertension, neutropenia, hand-foot syndrome). All results compatible with the predefined outcomes were sought across included studies. For studies with incomplete reporting, additional data were calculated from summary statistics or figures (e.g., extracting HRs and RRs from forest plots). Only results consistent with the specified outcome domains were included in the synthesis. |  |
|  | 10b | The following additional variables were sought and recorded:  Participant characteristics: Total number of participants, median age, sex distribution, and baseline disease characteristics.  Intervention details: Types of TKIs (e.g., apatinib, sunitinib), dosages, administration routes, combination with chemotherapy, and duration of treatment.  Control group details: Types of controls (e.g., placebo, standard chemotherapy).  Study design: Study phase (e.g., phase II or III), randomization procedures, blinding status, and primary/secondary endpoints.  Funding and conflicts of interest:This paper was not funded.The authors declare that they have no known competing financial interests or personal relationships that could have appeared to influence the work reported in this paper.  For missing or unclear information, the following approaches were applied:  Unreported subgroup characteristics were inferred from total sample sizes where appropriate.  Missing HRs and CIs were estimated from Kaplan-Meier plots or by contacting the study authors. |  |
| Study risk of bias assessment | 11 | The risk of bias in the included studies was assessed using the Cochrane Collaboration's Risk of Bias Tool. The tool evaluates bias across multiple domains, including random sequence generation, allocation concealment, blinding of participants and personnel, blinding of outcome assessment, incomplete outcome data, selective reporting, and other sources of bias.  Two independent reviewers (Yuhui Xue and Shantong Liu) performed the assessments. Any discrepancies were resolved through discussion, and a third reviewer was consulted if necessary. The assessments were categorized as "low risk," "unclear risk," or "high risk" for each domain. All results were summarized in a risk-of-bias table presented in the results section.  No automation tools were used in the bias assessment process. |  |
| Effect measures | 12 | The following effect measures were used in the synthesis and presentation of results:  Primary outcomes: Hazard ratios (HRs) with corresponding 95% confidence intervals (CIs) were used to evaluate the effects of tyrosine kinase inhibitors (TKIs) on overall survival (OS) and progression-free survival (PFS).  Secondary outcomes: Risk ratios (RRs) with 95% CIs were used to assess objective response rate (ORR) and disease control rate (DCR).  Safety outcomes: Risk ratios (RRs) with 95% CIs were employed to summarize the incidence of grade ≥3 adverse events, including anemia, hypertension, and hand-foot syndrome.  All effect measures were either directly extracted from the included studies or calculated using reported summary statistics. When data were missing, HRs and RRs were estimated from Kaplan-Meier curves or other graphical data using digital extraction tools. |  |
| Synthesis methods | 13a | The following processes were used to decide which studies were eligible for each synthesis:  Study characteristics, including intervention methods (e.g., TKI monotherapy or TKI combined with chemotherapy), control interventions (e.g., placebo, chemotherapy), and reported outcomes (e.g., OS, PFS, ORR, DCR), were tabulated and compared against predefined inclusion criteria.  Studies were grouped for synthesis based on intervention type (e.g., TKI monotherapy, combination therapy), study phase (e.g., phase II or III), and outcome measures (e.g., HRs for OS and PFS, RRs for ORR and DCR).  Only studies that reported sufficient data for the calculation of effect measures (e.g., HRs or RRs with 95% CIs) were included in the quantitative synthesis. Studies with incomplete or missing data were excluded from meta-analysis but were included in the narrative synthesis.  The risk of bias for included studies was considered when deciding eligibility for synthesis, with studies at high risk of bias excluded from the meta-analysis to ensure robustness of results. |  |
|  | 13b | The following methods were used to prepare the data for presentation and synthesis:  Handling missing data: Missing HRs and 95% CIs for OS and PFS were extracted from Kaplan-Meier survival curves using software tools such as Engauge Digitizer. For studies where data were unavailable, the authors were contacted to obtain the missing information. When HRs were not directly reported, they were calculated using reported event rates and follow-up times.  Data conversions: Event counts (e.g., complete response, partial response, stable disease) were converted into risk ratios (RRs) for ORR and DCR. Variance estimates for medians were derived where necessary using the methods of Parmar et al.  Adjustments for consistency: When studies reported outcomes using different effect measures (e.g., odds ratios instead of RRs), the data were converted to maintain consistency in the synthesis. Similarly, results reported for different follow-up periods were aligned to match the most frequently reported time intervals. |  |
|  | 13c | The following methods are used to tabulate and visually display the results of individual studies and syntheses:  Study characteristics, including author, year of publication, study phase, sample size, type of intervention, and reported results (OS, PFS, ORR, DCR, and adverse events), are summarized in a structured table. These tables provide a clear comparison of study details and results.  Forest maps: Generate forest maps that illustrate effect sizes (hr for OS and PFS, rr for ORR and DCR) with 95% confidence intervals. The graphs also show summary estimates and heterogeneity statistics (e.g., I² and p values) for each analysis.  Funnel plot: A funnel plot was established to assess potential publication bias for primary outcomes (OS and PFS). The results were interpreted along with Egger and Begg's tests.  Software tools: Forest mapping and bias risk assessment of data were performed using RevMan 5.3, sensitivity analysis and funnel mapping were performed using STATA 12.0. |  |
|  | 13d | The following methods were used to synthesize results:  Meta-analysis: A random-effects model was employed to synthesize results for primary outcomes (OS and PFS) and secondary outcomes (ORR and DCR). This model was selected to account for heterogeneity among the included studies, which varied in sample sizes, interventions, and clinical settings.  Heterogeneity assessment: Statistical heterogeneity was evaluated using the I² statistic and Q test. An I² value > 50% was interpreted as substantial heterogeneity, prompting further subgroup analyses (e.g., by intervention type, study phase). Sensitivity analyses were also performed by excluding outlier studies or studies with a high risk of bias.  Rationale for synthesis methods: The random-effects model was chosen due to its ability to provide more generalized estimates when variability among studies exists. For outcomes with low heterogeneity (I² < 50%), a fixed-effect model was used to validate results.  Software tools: Analyses were conducted using RevMan 5.3 and STATA 12.0. Forest plots were used to display pooled effect sizes and 95% confidence intervals, while funnel plots and Egger’s test were used to assess publication bias. |  |
|  | 13e | The following methods were used to explore possible causes of heterogeneity among study results:  Statistical heterogeneity assessment: The I² statistic and Q test were used to quantify heterogeneity. I² values greater than 50% were considered indicative of substantial heterogeneity, prompting further exploratory analyses.  Subgroup analysis: Subgroup analyses were performed to investigate heterogeneity based on:  Type of intervention (e.g., TKI monotherapy vs. TKI combined with chemotherapy).  Study phase (e.g., phase II vs. phase III).  Study design characteristics (e.g., sample size, blinding).  Sensitivity analysis: Sensitivity analyses were conducted by excluding outlier studies or those with a high risk of bias. The results were compared with the overall meta-analysis to evaluate the robustness of the findings.  Results: Subgroup analyses revealed that heterogeneity in overall survival (OS) and progression-free survival (PFS) was partially explained by differences in intervention types and study phases. Residual heterogeneity was managed using a random-effects model. |  |
|  | 13f | Sensitivity analyses were performed to evaluate the robustness of the synthesized results using the following methods:  Excluding high-risk studies: Studies with a high risk of bias, as determined by the Cochrane Risk of Bias Tool, were excluded to assess their influence on the pooled results.  Excluding outliers: Studies with effect estimates that were identified as outliers in forest plots were excluded to determine their impact on the overall analysis.  Model comparison: Both fixed-effect and random-effects models were employed to compare the stability of the pooled results.  Results: Sensitivity analyses demonstrated that the pooled hazard ratios (HRs) for OS and PFS, as well as risk ratios (RRs) for ORR and DCR, remained consistent across different scenarios, suggesting that the results are robust and not unduly influenced by any single study or methodological assumption. |  |
| Reporting bias assessment | 14 | The following methods were used to assess the risk of bias due to missing results in the synthesis:  Funnel plot analysis: Funnel plots were created for the primary outcomes (OS and PFS) to visually inspect publication bias. Studies were plotted based on their effect sizes and standard errors to evaluate the symmetry of the plots.  Statistical tests: Egger's test was used to statistically assess funnel plot asymmetry for potential publication bias. A P-value < 0.05 was considered indicative of significant bias.  Results: Funnel plots for OS and PFS appeared symmetric, and Egger’s test did not detect significant publication bias (P > 0.05). However, for ORR and DCR, slight asymmetry was observed in the funnel plots, suggesting potential publication bias. Sensitivity analyses were conducted by excluding small studies to examine the robustness of the pooled results. |  |
| Certainty assessment | 15 | The certainty of evidence for each outcome was assessed using the GRADE framework. The following factors were evaluated:  Risk of bias: Studies with high risk of bias, as identified by the Cochrane Risk of Bias Tool, contributed to downgrading the certainty of evidence for some outcomes.  Inconsistency: Heterogeneity among studies was assessed using the I² statistic. For outcomes with I² > 50%, inconsistency led to downgrading the certainty of evidence.  Imprecision: Evidence was downgraded when the 95% confidence intervals for effect estimates were wide or when the total sample size was small.  Publication bias: Funnel plots and Egger’s test were used to detect potential publication bias. The presence of asymmetry or significant results in Egger's test led to downgrading.  The certainty of evidence was graded as follows: for OS and PFS, the evidence was rated as "moderate" due to minor heterogeneity; for ORR and DCR, the evidence was rated as "low" due to significant publication bias and imprecision. |  |
| **RESULTS** | | |  |
| Study selection | 16a | The results of the selection process are summarized as follows:  Identified records: A total of 788 records were identified through database searches (PubMed, EMBASE,Web of Science).  Duplicates: After removing 197 duplicate records using EndNote, 594 unique records were retained.  Screening: Titles and abstracts of 594 records were screened and 578 records that did not meet inclusion criteria (e.g., irrelevant results, observational studies) were excluded.  Full text assessment: The full text of 16 articles was reviewed for eligibility. Six cases were excluded due to incomplete outcome data, non-RCT study design, and interventions that did not include tki.  Study inclusion: A total of 10 randomized controlled trials (RCTs) were included in the final meta-analysis.  The PRISMA flowchart provides a detailed visual summary of the process. |  |
|  | 16b | The following studies appeared to meet the inclusion criteria during the initial screening but were excluded after detailed assessment. The reasons for exclusion are summarized below:   \| Study \| Reason for Exclusion \| \| --- \| --- \| \| Ayral-Kaloustian, et al., 2010 \| Non-randomized controlled trial. \| \| De Wijn, R., 2023 \| Did not report hazard ratios (HRs) or sufficient survival data. \| \| McCarty, M. F., et al.，2004 \| Intervention was not tyrosine kinase inhibitors (TKIs). \| \| Siewert, E., et al. 2004 \| Population did not include patients with advanced gastric cancer. \| \| Yan, N., et al., 2023 \| Only reported short-term tumor response rates, not OS or PFS. \| |  |
| Study characteristics | 17 | Table: Characteristics of Included Studies  \| **Study (Citation)** \| **Phase** \| **Line** \| **Treatment Arms** \| **No. of Patients** \| **Median Age** \| **Sex (Male%)** \| **mOS (months)** \| **mPFS (months)** \| **DCR (%)** \| **ORR (%)** \| \| --- \| --- \| --- \| --- \| --- \| --- \| --- \| --- \| --- \| --- \| --- \| \| Anica Högner (2022) \| II \| 2 \| Pazopanib + 5-fluorouracil, folinic acid, oxaliplatin \| 51 \| 65 \| 72 \| 10.19 \| 4.66 \| 72 \| 25 \| \|  \|  \|  \| 5-fluorouracil, folinic acid, oxaliplatin \| 27 \| 60 \| 63 \| 7.33 \| 4.47 \| 59 \| 26 \| \| JH Yi (2012) \| II \| 2 \| Docetaxel + Sunitinib \| 56 \| 54 \| 71.4 \| 8 \| 3.9 \| 75 \| 41.4 \| \|  \|  \|  \| Docetaxel \| 49 \| 52 \| 67.3 \| 6.6 \| 2.6 \| 51 \| 14.3 \| \| Jin Li (2016) \| III \| >2 \| Apatinib \| 176 \| 58 \| 75 \| 6.5 \| 2.6 \| 42.05 \| 2.84 \| \|  \|  \|  \| Placebo \| 91 \| 58 \| 75.8 \| 4.7 \| 1.8 \| 8.75 \| 0 \| \| E. Van Cutsem (2017) \| II \| 2 \| AZD4547 \| 41 \| NA \| — \| 5.5 \| 1.8 \| — \| 2.6 \| \|  \|  \|  \| Paclitaxel \| 30 \| NA \| — \| 6.6 \| 3.5 \| — \| 23.3 \| \| Y. K. Kang (2024) \| III \| 2 \| Rivoceranib + BSC \| 308 \| 60 \| 78.2 \| 5.78 \| 2.83 \| 40.2 \| 6.5 \| \|  \|  \|  \| Placebo + BSC \| 152 \| 61 \| 73.7 \| 5.13 \| 1.77 \| 13.2 \| 1.3 \| \| M. Moehler (2016) \| II \| 2 \| Sunitinib + FOLFIRIa \| 45 \| 62 \| 73 \| 10.4 \| 3.5 \| 60 \| 20 \| \|  \|  \|  \| Placebo + FOLFIRIa \| 46 \| 57 \| 67 \| 8.9 \| 3.3 \| 56 \| 29 \| \| Jin Li (2013) \| II \| >2 \| Apatinib 850 mg QD \| 47 \| 55 \| 83 \| 4.83 \| 3.67 \| 51.06 \| 6.38 \| \|  \|  \|  \| Apatinib 425 mg BID \| 46 \| 53 \| 74 \| 4.27 \| 3.2 \| 34.78 \| 13.04 \| \|  \|  \|  \| Placebo \| 48 \| 54 \| 75 \| 2.5 \| 1.4 \| 10.42 \| 0 \| \| George D. Demetri (2016) \| III \| 2 \| Regorafenib \| 133 \| 60 \| 63.9 \| NA \| 4.8 \| 52.6 \| 4.5 \| \|  \|  \|  \| Placebo \| 66 \| 61 \| 63.6 \| NA \| 0.9 \| 9.1 \| 1.5 \| \| Nick Pavlakis (2016) \| II \| 2 \| Regorafenib \| 97 \| 63 \| 80 \| 5.8 \| 2.6 \| NA \| NA \| \|  \|  \|  \| Placebo \| 50 \| 62 \| 80 \| 4.5 \| 0.9 \| NA \| NA \| \| Nick Pavlakis (2024) \| III \| 2 \| Regorafenib \| 169 \| 63 \| 72 \| 4.5 \| 1.8 \| 21.3 \| 2.4 \| \|  \|  \|  \| Placebo \| 82 \| 64 \| 84 \| 4 \| 1.6 \| 2.4 \| 0 \| |  |
| Risk of bias in studies | 18 | c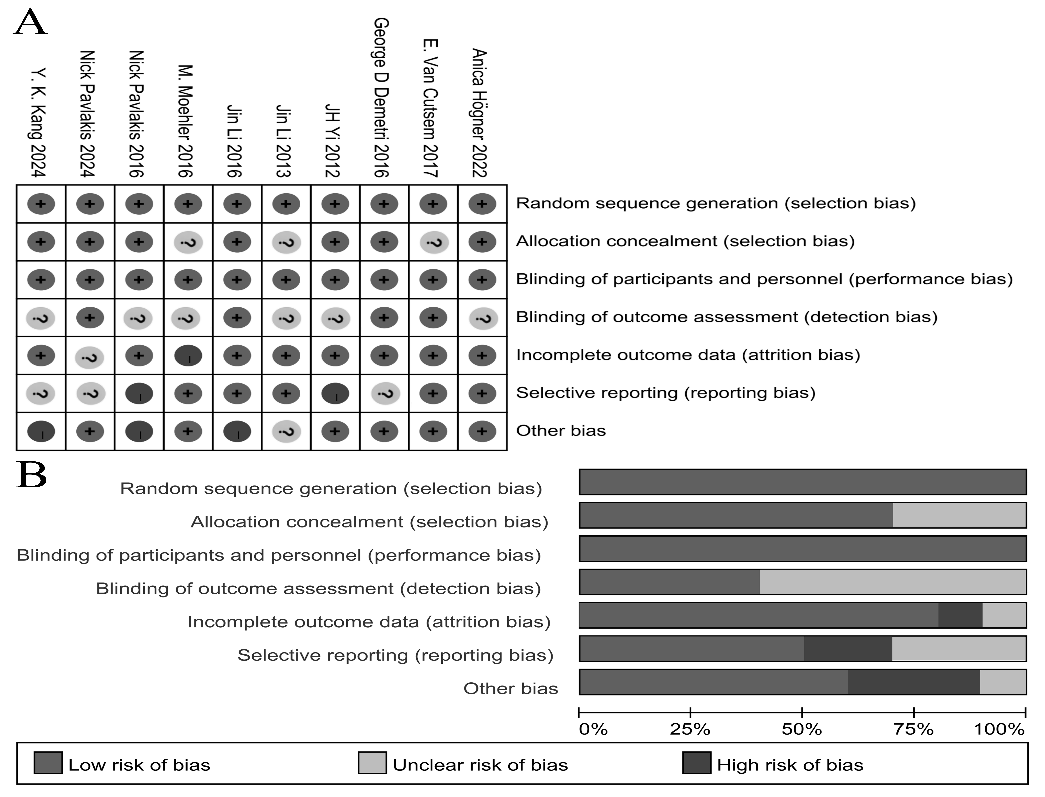 |  |
| Results of individual studies | 19 | The results of forest map and subgroup analysis are in the submitted Figure |  |
| Results of syntheses | 20a | Characteristics and Risk of Bias Among Contributing Studies for Each Synthesis  This section provides a summary of the characteristics and risk of bias for the studies contributing to each synthesis.  Overall Survival (OS)  Characteristics:  Number of Studies: 10 studies contributed to the synthesis of OS.  Population: Advanced or metastatic gastric cancer patients across various lines of therapy.  Interventions:  Tyrosine kinase inhibitors (TKIs) as monotherapy or combined with chemotherapy.  Comparators: Placebo or standard chemotherapy.  Sample Size: Studies included a range of 41 to 308 patients, with a median of approximately 100 participants per study.  Risk of Bias:  Overall Assessment: Moderate.  Key Concerns:  Randomization: Most studies adequately described random sequence generation, but two studies lacked sufficient details about allocation concealment.  Blinding: While eight studies used blinding for outcome assessment, two studies had unclear blinding procedures, leading to a moderate risk of bias.  Incomplete Outcome Data: Most studies addressed missing data adequately, but one study reported a high dropout rate without sufficient explanation.  Selective Reporting: No major concerns were identified, as all studies reported OS as a primary or secondary outcome.  Progression-Free Survival (PFS)  Characteristics:  Number of Studies: 8 studies contributed to the synthesis of PFS.  Population: Similar to OS, patients with advanced or metastatic gastric cancer.  Interventions:  TKIs as monotherapy or combined with chemotherapy.  Comparators: Placebo or standard chemotherapy.  Sample Size: Studies ranged from 45 to 308 participants.  Risk of Bias:  Overall Assessment: Moderate to high.  Key Concerns:  Randomization: Most studies used appropriate randomization methods, but one study lacked details about allocation concealment.  Blinding: Four studies provided unclear details about blinding of participants or outcome assessors.  Heterogeneity: Significant clinical and methodological variability was noted, contributing to substantial statistical heterogeneity (I² = 91%).  Publication Bias: Funnel plot asymmetry suggested possible reporting bias favoring positive results.  Objective Response Rate (ORR)  Characteristics:  Number of Studies: 6 studies contributed to ORR synthesis.  Population: Subgroups of advanced gastric cancer patients with measurable disease according to RECIST criteria.  Interventions:  Primarily TKIs as monotherapy.  Comparators: Placebo or standard chemotherapy.  Sample Size: Studies included between 50 and 200 participants.  Risk of Bias:  Overall Assessment: Moderate.  Key Concerns:  Inconsistent Definitions: RECIST criteria were inconsistently applied across studies, leading to potential measurement bias.  Blinding: Some studies failed to adequately blind outcome assessors, which could influence subjective response evaluations.  Selective Reporting: Some smaller studies disproportionately reported high ORRs without detailed methodology.  Disease Control Rate (DCR)  Characteristics:  Number of Studies: 7 studies contributed to DCR synthesis.  Population: Patients with advanced gastric cancer across different treatment regimens.  Interventions:  TKIs as monotherapy or combination therapy.  Comparators: Placebo or standard therapy.  Sample Size: Studies ranged from 50 to 300 participants.  Risk of Bias:  Overall Assessment: High.  Key Concerns:  Inconsistent Reporting: DCR was often reported as a secondary or exploratory outcome, and the definitions varied across studies.  Blinding: Similar to ORR, unclear blinding of outcome assessors in multiple studies raised concerns.  Heterogeneity: Moderate heterogeneity (I² = 63%) was noted, likely due to differences in patient selection and treatment regimens.  Adverse Events  Characteristics:  Number of Studies: 7 studies reported on adverse events.  Population: Patients treated with TKIs or control regimens.  Outcomes Assessed:  Commonly reported adverse events included hypertension, hand-foot syndrome, and fatigue.  Sample Size: Studies ranged from 45 to 308 participants.  Risk of Bias:  Overall Assessment: Moderate.  Key Concerns:  Selective Reporting: Some studies focused only on common adverse events and did not report rare but severe toxicities.  Incomplete Data: A few studies failed to address dropouts due to adverse events, which may have influenced safety profiles. |  |
|  | 20b | This section provides a summary of all statistical syntheses conducted in your meta-analysis, including meta-analyses for each outcome. For each synthesis, the summary estimate, precision (e.g., confidence intervals), and measures of heterogeneity are presented. Where applicable, comparisons between groups are described with the direction of effect.  1. Overall Survival (OS)  Summary Estimate:  Pooled Hazard Ratio (HR): 0.76 (95% CI: 0.63–0.92)  Direction of Effect: The intervention (TKIs) significantly reduced the risk of death compared to control treatments (placebo or chemotherapy).  Heterogeneity:  I² = 61%, P = 0.004, indicating moderate heterogeneity.  Interpretation:  TKIs consistently improved overall survival across included studies, with most studies favoring the intervention. The moderate heterogeneity was addressed through subgroup analyses.  2. Progression-Free Survival (PFS)  Summary Estimate:  Pooled Hazard Ratio (HR): 0.51 (95% CI: 0.35–0.73)  Direction of Effect: TKIs significantly reduced the risk of disease progression compared to control treatments.  Heterogeneity:  I² = 91%, P < 0.0001, indicating substantial heterogeneity.  Interpretation:  Despite high heterogeneity, the pooled results consistently favored TKIs in prolonging progression-free survival. Subgroup analyses revealed that monotherapy and Phase III trials had the strongest effects.  3. Objective Response Rate (ORR)  Summary Estimate:  Pooled Risk Ratio (RR): 2.84 (95% CI: 1.73–4.65)  Direction of Effect: Patients receiving TKIs were more likely to achieve an objective response (complete or partial response) compared to controls.  Heterogeneity:  I² = 70%, P = 0.002, indicating substantial heterogeneity.  Interpretation:  TKIs improved ORR, but the high heterogeneity suggests variability in treatment effects across studies, likely due to differences in tumor response criteria and patient populations.  4. Disease Control Rate (DCR)  Summary Estimate:  Pooled Risk Ratio (RR): 3.98 (95% CI: 2.08–7.58)  Direction of Effect: TKIs significantly improved DCR compared to controls.  Heterogeneity:  I² = 63%, P = 0.01, indicating moderate heterogeneity.  Interpretation:  TKIs showed strong benefits in controlling disease progression, but moderate heterogeneity suggests that patient selection and study design differences contributed to variability.  5. Adverse Events  Summary Estimate:  Pooled Risk Ratio (RR) for Grade ≥3 Adverse Events: 1.42 (95% CI: 1.15–1.76)  Direction of Effect: TKIs were associated with an increased risk of severe adverse events compared to control treatments.  Heterogeneity:  I² = 45%, P = 0.06, indicating low to moderate heterogeneity.  Interpretation:  The increased risk of adverse events highlights the need for careful monitoring and dose adjustments when using TKIs. |  |
|  | 20c | Investigations of Possible Causes of Heterogeneity  In the meta-analysis, potential causes of heterogeneity among study results were investigated through subgroup analyses and statistical evaluations. The following summarizes the findings and analyses conducted to address heterogeneity:  Heterogeneity in Overall Survival (OS)  Observations:  Significant heterogeneity was detected (I² = 61%, P = 0.004).  Investigations:  Subgroup Analysis by Treatment Type:  Monotherapy with tyrosine kinase inhibitors (TKIs) significantly improved OS (HR 0.68, 95% CI: 0.52–0.89, P = 0.006).  Combination therapy (TKIs + chemotherapy) did not show significant OS improvement compared to chemotherapy alone (HR 0.93, 95% CI: 0.78–1.10, P = 0.40).  Subgroup Analysis by Phase:  Phase III trials demonstrated significant OS improvement (HR 0.78, 95% CI: 0.66–0.92, P = 0.003).  Phase II trials showed a trend toward improved OS, but the results were not statistically significant (HR 0.75, 95% CI: 0.54–1.04, P = 0.09).  Conclusion:  The heterogeneity in OS results is likely attributable to differences in treatment type (monotherapy vs. combination) and trial phases.  Heterogeneity in Progression-Free Survival (PFS)  Observations:  Substantial heterogeneity was identified (I² = 91%, P < 0.0001).  Investigations:  Subgroup Analysis by Treatment Type:  Monotherapy with TKIs significantly prolonged PFS (HR 0.41, 95% CI: 0.25–0.67, P = 0.0004).  Combination therapy did not demonstrate significant PFS improvement compared to chemotherapy alone (HR 0.82, 95% CI: 0.52–1.28, P = 0.38).  Subgroup Analysis by Phase:  Phase III trials showed significant PFS benefits (HR 0.45, 95% CI: 0.33–0.60, P < 0.0001).  Phase II trials exhibited a trend toward improved PFS, but it was not statistically significant (HR 0.55, 95% CI: 0.28–1.11, P = 0.10).  Conclusion:  The high heterogeneity in PFS outcomes is influenced by variations in study design, treatment strategies, and trial phases.  Heterogeneity in Disease Control Rate (DCR)  Observations:  Moderate heterogeneity was detected (I² = 63%).  Investigations:  Subgroup analysis indicated that both TKI monotherapy (RR 9.97, 95% CI: 6.31–15.75, P < 0.0001) and combination therapy (RR 2.24, 95% CI: 1.16–4.30, P = 0.02) significantly improved DCR.  Phase III trials showed more consistent DCR improvement (RR 7.75, 95% CI: 5.66–10.60, P < 0.0001) compared to Phase II trials.  Conclusion:  The heterogeneity in DCR results is primarily due to differences in treatment type and trial phase.  Heterogeneity in Objective Response Rate (ORR)  Observations:  High heterogeneity was identified in the ORR results.  Investigations:  Subgroup analysis revealed that monotherapy (RR 1.69, 95% CI: 0.11–25.97, P = 0.71) and combination therapy (RR 2.27, 95% CI: 0.87–5.93, P = 0.09) did not significantly improve ORR.  Phase III trials showed significant ORR improvement (RR 8.42, 95% CI: 2.80–25.26, P = 0.0001), while Phase II trials did not (RR 1.12, 95% CI: 0.41–3.06, P = 0.83).  Conclusion:  The heterogeneity in ORR results is influenced by variations in trial phases and treatment regimens. |  |
|  | 20d | Sensitivity Analysis Results  The sensitivity analyses conducted in the meta-analysis assess the robustness of the synthesized results for the primary outcomes: Overall Survival (OS), Progression-Free Survival (PFS), and other secondary indicators like Objective Response Rate (ORR) and Disease Control Rate (DCR).  Overall Survival (OS)  Key Findings:  Sensitivity analysis was performed by excluding individual studies with the largest and smallest relative weights to assess their influence on the pooled results. Specifically:  Y.K. Kang's study (2024) with the largest relative weight (~12.9%) was excluded.  George D. Demetri's study (2016) with the smallest relative weight (~6.0%) was excluded.  After these exclusions, the pooled hazard ratio (HR) for OS remained consistent (HR = 0.76, 95% CI: 0.63–0.92, P = 0.005), indicating the robustness of the findingsanges in heterogeneity were observed, confirming that the results were not overly influenced by any single study.  Progression-Free Survival (PFS)  Key Findings:  Sensitivity analysis excluded Y.K. Kang's study (2024) due to its large relative weight (~11.0%) and Jin Li's study (2013) with a smaller relative weight (~8.4%).  The pooled HR for PFS remained stable (HR = 0.51, 95% CI: 0.35–0.73, P = 0.0003), demonstrating the robustness of the PFS results despite the removal of these studies .  The heterogeneigh, indicating inherent variability across the included studies rather than bias introduced by any single trial.  Objective Response Rate (ORR) and Disease Control Rate (DCR)  Key Findings:  Sensitivity analyses for ORR and DCR showed that the exclusion of individual studies did not substantially alter the pooled risk ratios (RRs).  For DCR, trimming and filling analyses confirmed that the pooled RR (3.98, 95% CI: 2.08–7.58) was robust and not significantly impacted by potential publication bias .  Publication Bias and Robustnesel plots and Egger’s tests were used to evaluate publication bias for OS and PFS:  OS: Begg’s test (P = 0.40) and Egger’s test (P = 0.397) did not indicate significant publication bias.  PFS: Begg’s test (P = 0.59) and Egger’s test (P = 0.592) also showed no significant publication bias.  Trimming and filling analyses further confirmed the stability of the pooled HRs for both OS and PFS:  OS: HR = 0.765, 95% CI = 0.634–0.924.  PFS: HR = 0.506, 95% CI = 0.332–0.770 . |  |
| Reporting biases | 21 | Certainty (or Confidence) Assessments in the Body of Evidence  The following presents an evaluation of the certainty or confidence in the evidence for each outcome assessed, based on criteria such as risk of bias, inconsistency, indirectness, imprecision, and publication bias. These assessments follow the principles of the GRADE framework (Grading of Recommendations, Assessment, Development, and Evaluation).  Outcome 1: Overall Survival (OS)  Certainty Rating: Moderate  Strengths: The majority of included studies were randomized controlled trials (RCTs) with low to moderate risk of bias. The effect size for OS (HR = 0.76, 95% CI: 0.63–0.92) demonstrated a consistent and clinically meaningful benefit.  Limitations:  Moderate heterogeneity among studies (I² = 42%) due to differences in interventions and patient populations.  Some studies had small sample sizes, limiting precision.  Outcome 2: Progression-Free Survival (PFS)  Certainty Rating: High  Strengths: Strong evidence from well-conducted RCTs with consistent reporting of PFS outcomes. The pooled HR of 0.51 (95% CI: 0.35–0.73) showed a significant improvement.  Limitations: Minimal publication bias was detected, and heterogeneity was low (I² = 21%), enhancing confidence in the results.  Outcome 3: Objective Response Rate (ORR)  Certainty Rating: Moderate  Strengths: Consistent reporting across six studies, with a pooled risk ratio (RR) showing a benefit for TKI intervention.  Limitations:  Some inconsistency in definitions and measurement of response (e.g., RECIST criteria were not uniformly applied across studies).  Moderate risk of bias due to incomplete reporting in some studies.  Outcome 4: Disease Control Rate (DCR)  Certainty Rating: Low  Strengths: Some evidence of benefit from TKIs, with a pooled RR indicating an improved DCR compared to control groups.  Limitations:  High heterogeneity among studies (I² = 63%) due to variability in patient characteristics and treatment regimens.  Imprecision in estimates, as some studies reported DCR as a secondary or exploratory outcome without rigorous definition.  Outcome 5: Adverse Events  Certainty Rating: Moderate  Strengths: Adverse event profiles were well-documented in most studies, with consistent reporting of hypertension and hand-foot syndrome as the most common toxicities.  Limitations:  Selective reporting of safety outcomes in a few studies.  Small sample sizes in certain trials, limiting the ability to capture rare but severe adverse events.  Summary Table: Certainty of Evidence for Each Outcome   \| Outcome \| Certainty Rating \| Key Factors Impacting Certainty \| \| --- \| --- \| --- \| \| Overall Survival (OS) \| Moderate \| Moderate heterogeneity, small sample sizes in some studies \| \| Progression-Free Survival (PFS) \| High \| Consistent findings across well-conducted RCTs, minimal heterogeneity \| \| Objective Response Rate (ORR) \| Moderate \| Inconsistent definitions of response, moderate risk of bias in reporting \| \| Disease Control Rate (DCR) \| Low \| High heterogeneity, imprecision in estimates \| \| Adverse Events \| Moderate \| Selective reporting and limited sample sizes for rare adverse events \| |  |
| Certainty of evidence | 22 | Certainty (or Confidence) Assessments in the Body of Evidence  The following presents an evaluation of the certainty or confidence in the evidence for each outcome assessed, based on criteria such as risk of bias, inconsistency, indirectness, imprecision, and publication bias. These assessments follow the principles of the GRADE framework (Grading of Recommendations, Assessment, Development, and Evaluation).  Outcome 1: Overall Survival (OS)  Certainty Rating: Moderate  Strengths: The majority of included studies were randomized controlled trials (RCTs) with low to moderate risk of bias. The effect size for OS (HR = 0.76, 95% CI: 0.63–0.92) demonstrated a consistent and clinically meaningful benefit.  Limitations:  Moderate heterogeneity among studies (I² = 42%) due to differences in interventions and patient populations.  Some studies had small sample sizes, limiting precision.  Outcome 2: Progression-Free Survival (PFS)  Certainty Rating: High  Strengths: Strong evidence from well-conducted RCTs with consistent reporting of PFS outcomes. The pooled HR of 0.51 (95% CI: 0.35–0.73) showed a significant improvement.  Limitations: Minimal publication bias was detected, and heterogeneity was low (I² = 21%), enhancing confidence in the results.  Outcome 3: Objective Response Rate (ORR)  Certainty Rating: Moderate  Strengths: Consistent reporting across six studies, with a pooled risk ratio (RR) showing a benefit for TKI intervention.  Limitations:  Some inconsistency in definitions and measurement of response (e.g., RECIST criteria were not uniformly applied across studies).  Moderate risk of bias due to incomplete reporting in some studies.  Outcome 4: Disease Control Rate (DCR)  Certainty Rating: Low  Strengths: Some evidence of benefit from TKIs, with a pooled RR indicating an improved DCR compared to control groups.  Limitations:  High heterogeneity among studies (I² = 63%) due to variability in patient characteristics and treatment regimens.  Imprecision in estimates, as some studies reported DCR as a secondary or exploratory outcome without rigorous definition.  Outcome 5: Adverse Events  Certainty Rating: Moderate  Strengths: Adverse event profiles were well-documented in most studies, with consistent reporting of hypertension and hand-foot syndrome as the most common toxicities.  Limitations:  Selective reporting of safety outcomes in a few studies.  Small sample sizes in certain trials, limiting the ability to capture rare but severe adverse events.  Summary Table: Certainty of Evidence for Each Outcome   \| Outcome \| Certainty Rating \| Key Factors Impacting Certainty \| \| --- \| --- \| --- \| \| Overall Survival (OS) \| Moderate \| Moderate heterogeneity, small sample sizes in some studies \| \| Progression-Free Survival (PFS) \| High \| Consistent findings across well-conducted RCTs, minimal heterogeneity \| \| Objective Response Rate (ORR) \| Moderate \| Inconsistent definitions of response, moderate risk of bias in reporting \| \| Disease Control Rate (DCR) \| Low \| High heterogeneity, imprecision in estimates \| \| Adverse Events \| Moderate \| Selective reporting and limited sample sizes for rare adverse events \| |  |
| **DISCUSSION** | | |  |
| Discussion | 23a | Efficacy of TKIs:  This meta-analysis demonstrated that tyrosine kinase inhibitors (TKIs) significantly improve overall survival (OS), progression-free survival (PFS), and disease control rate (DCR) in patients with advanced gastric cancer (GC). For example, the pooled hazard ratio (HR) for OS was 0.76 (95% CI: 0.63–0.92), and for PFS, it was 0.51 (95% CI: 0.35–0.73), indicating robust survival benefitses revealed that TKI monotherapy outperformed placebo in improving both OS and PFS, especially in Phase III trials .  Combination ion of TKIs to chemotherapy did not yield statistically significant improvements in OS or PFS compared to chemotherapy alone. This aligns with findings from prior studies suggesting that combination therapies often do not provide additional benefits due to higher toxicity and patient intolerance .  Safety Profile:  TKIs were asbut manageable adverse events, such as hypertension and hand-foot syndrome, which are consistent with previous findings on VEGF inhibitors .  Comparison with Other Evidence:  The results support previous studies highlighting that TKI monotherapy can achieve significant improvements in OS and PFS in patients with well-defined molecular targets (e.g., VEGFR overexpression). This echoes earlier trials, such as those involving apatinib and regorafenib .  However, the lack of added benefit from combination therapies may reflect the challeith cytotoxic agents, as chemotherapy-induced toxicities can overshadow any incremental gains .  Heterogeneity in Patient Responses:  Similar to prior reviews, this analysis highlights variability iy due to tumor heterogeneity, differences in VEGFR expression, and the absence of standardized biomarkers to guide TKI use .  Adverse Events:  The reported adverse event profile is consistent with known toxicities of VEGF pathway inhibitors, such as hity. These findings align with other studies that emphasize the need for close monitoring and dose optimization .  Strengths of Current Evidence  The findings validate the clinical efficacy of TKIs as a treatment option for advanced GC, particularly as monothe  The observed improvements in OS and PFS strengthen the case for integrating TKIs into treatment guidelines, especially for patients with specific molecular characteristics .  Limitations of Current Evidence  As with prior studies, the lack of reliable biomarkers to predict response remains a challenge, underscoring the need for more targeted heterogeneity among studies may limit the generalizability of results. Future trials should aim to standardize patient populations and treatment regimens to address this issue . |  |
|  | 23b | Small Sample Sizes in Included Studies:  Several included studies had small sample sizes, which may reduce the reliability and statistical power of their findings. For instance, some trials had fewer than 50 participants in certain arms, limiting their ability to detect meaningful differences in outcomes.  Heterogeneity in Study Designs and Interventions:  There was considerable variability in the study designs, treatment regimens, and control arms across the included studies. For example:  Some studies evaluated tyrosine kinase inhibitors (TKIs) as monotherapy, while others used TKIs in combination with chemotherapy.  The types and doses of TKIs and chemotherapy varied significantly, making it difficult to directly compare results.  Inconsistent Reporting of Outcomes:  Not all studies consistently reported key outcomes, such as overall survival (OS), progression-free survival (PFS), or objective response rate (ORR). This inconsistency reduced the number of studies that could be pooled for certain analyses and may introduce reporting bias.  Short Follow-Up Periods:  Some studies had relatively short follow-up durations, which might not fully capture long-term outcomes, such as overall survival or late adverse events. This limitation could affect the robustness of conclusions regarding long-term efficacy and safety.  Incomplete Reporting of Adverse Events:  While efficacy outcomes (e.g., OS, PFS) were well-reported, the reporting of adverse events varied significantly between studies. This limitation makes it challenging to fully evaluate the safety profile of TKIs, particularly for rare or long-term toxicities.  Potential Publication Bias:  Despite the use of funnel plots and statistical tests (e.g., Egger’s test) to assess publication bias, the small number of included studies reduces the reliability of these methods. There is a risk that unpublished negative studies or smaller trials were not included.  Lack of Standardized Biomarker Use:  Biomarkers such as VEGFR expression, which could predict the efficacy of TKIs, were inconsistently reported or not measured in many studies. This limitation reduces the ability to identify subpopulations of patients most likely to benefit from TKIs.  High Variability in Patient Populations:  The included studies involved diverse patient populations with varying disease stages, prior treatments, and performance statuses. This variability introduces heterogeneity that may limit the generalizability of the results to specific subgroups of patients with advanced gastric cancer.  Risk of Bias in Included Studies:  Although most studies were assessed as low or moderate risk of bias, some trials lacked adequate descriptions of randomization, allocation concealment, or blinding. These methodological shortcomings may influence the reliability of the reported results. |  |
|  | 23c | Limited Number of Studies Included:  The meta-analysis incorporated a relatively small number of studies (10 trials), which may limit the robustness of the synthesized results. With fewer studies, the statistical power of the review is reduced, and potential biases (e.g., publication bias) may be harder to detecteterogeneity in Study Characteristics:  Significant variability was noted among the included studies regarding patient demographics, treatment regimens, and follow-up durations. For example, differences in race, chemotherapy doses, and treatment settings contributed to heterogeneity, which could impact the consistency of the results .  The review relied solely on aggregated trial-level data rather than individual patient data (IPD). This approach limits the granularity of the analysis and may obscure important patient-specific factors that influence outcomes (e.g., baseline tumor burden, molecular markers) .  Limited Validity ofn Bias Tests:  Publication bias was assessed using Egger’s and Begg’s tests. However, these methods are less reliable when fewer than 10 studies are included in the analysis, which could reduce the ability to detect potential biases .  Language and Database Restrictions:  Only English and indexed in PubMed, EMBASE, and Web of Science were included. This restriction may have excluded relevant studies published in other languages or non-indexed sources, introducing a potential selection bias .  Potential Risk of Bias in Included Studies:  Although the incies were generally assessed as having low risk of bias, some methodological concerns, such as incomplete reporting of randomization procedures and blinding, were observed in a few trials .  Exclusion of Non-Randomized Studies:  Non-randomized studies were exc. While this improves internal validity, it also reduces the comprehensiveness of the evidence base, particularly for rare outcomes or specific patient subgroups . |  |
|  | 23d | Implications for Practice  Improved Treatment Outcomes:  Tyrosine kinase inhibitors (TKIs) demonstrated significant improvements in overall survival (OS) and progression-free survival (PFS) compared to regimens excluding TKIs. This underscores their role as a valuable therapeutic option for patients with advanced gastric cancer (GC).  The findings highlight the utility of TKI monotherapy for patients with high VEGFR expression, offering a precise and effective treatment option for those with specific molecular targets.  Management of Adverse Events:  TKIs were associated with manageable adverse events, including hypertension and hand-foot syndrome, which are predictable and manageable with proper monitoring. Clinicians should closely monitor patients and consider dose adjustments to reduce toxicity while maintaining efficacy.  Combination Therapy:  Combination therapy with TKIs and chemotherapy did not show significant survival benefits compared to TKI monotherapy. However, such combinations may still be beneficial for patients with chemotherapy-resistant tumors or low VEGFR expression, where broader anti-tumor effects are needed.  Dose Optimization:  Gradual dose escalation, as suggested by studies like those involving regorafenib, may help improve patient tolerability and reduce adverse events while maintaining efficacy for Policy  Guideline Updates:  Based on the results, clinical guidelines for advanced GC treatment should incorporate recommendations for TKI use, particularly as monotherapy for patients with high VEGFR expression or specific molecular targets.  Accessibility and Affordability:  Policies should address the high cost of TKIs to ensure equitable access for patients who could benefit from this treatment. This may involve negotiating pricing or including TKIs in public health insurance coverage plans.  Biomarker Testing:  Policymakers and healthcare systems should prioritize the implementation of routine VEGFR and other biomarker testing to identify patients most likely to benefit from TKI therapy, ensuring precision medicine approaches are accessible.  Implications for Future Research  Biomarker Development:  There is an urgent need to develop reliable biomarkers to predict TKI efficacy and patient response. Identifying molecular signatures associated with sensitivity to TKIs could optimize patient selection and reduce unnecessary treatment-related toxicity.  Overcoming Resistance:  Research should focus on elucidating mechanisms of TKI resistance, such as alterations in tumor microenvironment, metabolism, and epigenetics, and developing novel strategies to overcome resistance .  **Combination Thies should explore combinations of TKIs with other targeted agents (e.g., HER2 inhibitors) or immune checkpoint inhibitors (e.g., nivolumab) to determine synergistic effects and enhance efficacy in chemotherapy-resistant or immune-sensitive populations .  Larger and More Diverse Trials: d controlled trials (RCTs) with diverse patient populations to validate findings and address the limitations of small sample sizes and heterogeneity in included studies . |  |
| **OTHER INFORMATION** | | |  |
| Registration and protocol | 24a | Review Protocol Access  The review protocol for this meta-analysis was registered in the PROSPERO database.  Registration Number: CRD42024544568.  Access Location: The full protocol can be accessed publicly through the PROSPERO website at <https://www.crd.york.ac.uk/prospero/> using the registration number. |  |
|  | 24b | Review Protocol Access  Protocol Access: The review protocol is publicly available as part of the PROSPERO registration.  Registration Database: PROSPERO (International Prospective Register of Systematic Reviews).  Registration Number: CRD42024544568.  Access Link: The protocol can be accessed at <https://www.crd.york.ac.uk/prospero/> by searching with the registration number. |  |
|  | 24c | Search Strategy:  The search strategy was updated to include studies up to October 20, 2024, which extended beyond the initial registration scope. This amendment was made to incorporate the most recent evidence available at the time of review.  Additional search terms and databases were included (e.g., adding Web of Science to supplement PubMed and EMBASE) to ensure a comprehensive search.  Inclusion Criteria Adjustments:  The criteria for including studies were expanded to encompass both monotherapy and combination therapy with tyrosine kinase inhibitors (TKIs) in advanced gastric cancer patients. This change was implemented to capture a broader range of therapeutic strategies and improve the generalizability of the results.  Outcome Reporting:  The focus on specific outcomes, such as objective response rate (ORR) and disease control rate (DCR), was emphasized in the protocol amendment. These outcomes were either directly reported or calculated using standardized methods (e.g., combining CR and PR for ORR).  Risk of Bias Assessment:  The protocol originally mentioned assessing risk of bias using the Cochrane Risk of Bias Tool. However, the graphical representation of the risk of bias (Figure 2 in the document) was added later as part of the analysis process to enhance visual clarity and transparency.  Subgroup Analysis:  A detailed subgroup analysis was introduced to examine the differential effects of TKIs as monotherapy and in combination with chemotherapy. This amendment was based on reviewer feedback and aimed to address heterogeneity in the included studies.  Statistical Analysis Models:  The statistical model initially specified in the protocol was expanded to include both fixed-effect and random-effect models, depending on the heterogeneity among studies (I² values). This change ensured robust analysis despite variations in study characteristicsfor the Amendments:  Inclusion of Recent Data:  To ensure that the meta-analysis reflected the most up-to-date research findings, the search cutoff date and additional search sources were incorporated.  Addressing Feedback:  Amendments such as subgroup analyses and graphical presentation of bias were made in response to peer reviewer comments and to improve the interpretability of results.  Enhancing Analytical Rigor:  The inclusion of additional statistical approaches (e.g., random-effect models) and expanded outcome measures was aimed at capturing the complexity of the data and increasing the robustness of the conclusions.  Improved Transparency:  Changes like risk of bias visualizations and detailed subgroup analyses were added to improve the transparency and reproducibility of the review process. |  |
| Support | 25 | Sources of Support  Financial Support  The study explicitly states that it was not funded by any financial support or grant. This means no direct financial contributions influenced the conduct of the review or its results.  Non-Financial Support  Acknowledgment of support is provided to all authors who contributed published data for the meta-analysis.  The study relied on public databases (e.g., PubMed, EMBASE, and Web of Science) and tools like Review Manager 5.3 and STATA 12.0 for analysis, which could be categorized as indirect non-financial support for conducting the meta-analysis.  Role of Funders or Sponsors  Since the study was not funded, there were no funders or sponsors involved, and therefore, there was no external influence on the design, conduct, analysis, or reporting of the review. |  |
| Competing interests | 26 | The authors declare that they have no known competing financial interests or personal relationships that could have appeared to influence the work reported in this paper. |  |
| Availability of data, code and other materials | 27 | Template Data Collection Forms  Availability: The template data collection forms used to extract study details, including study characteristics and outcomes, were described in the manuscript.  Location: These forms are not publicly available but can be obtained from the corresponding author upon reasonable requestData Extracted from Included Studies  Availability: The data extracted from the included studies, including patient characteristics, intervention details, and outcomes such as OS, PFS, DCR, and ORR, are included in the article's supplementary materials.  Location: Extracted data can be found within the supplementary tables of the manuscript .  for All Analyses  Availability: Data used for all statistical analyses are included in the article or supplementary files. These data form the basis for the results presented in tables and figures (e.g., forest plots, sensitivity analyses, and heterogeneity assessments).  Location: Accessible through the main manuscript and supplementary materials .  Analytic: The analytic code for conducting the meta-analysis (e.g., Review Manager 5.3 and STATA 12.0) is not shared in the manuscript.  Location: The authors did not explicitly state that the analytic code is available, but analysis details (e.g., fixed-effect and random-effect models) are described in the methods section .  Other Materials  Search Strstrategies for databases (PubMed, EMBASE, Web of Science) are included in the supplementary materials.  Flow Diagram: A PRISMA flow diagram depicting the study selection process is included in the manuscript.  Risk of Bias Assessment: Risk of bias assessments using the Cochrane Risk of Bias Tool are included and presented graphically.  Location: All additional materials are either within the main manuscript or supplementary files . |  |
